# Membrane tension increases fusion efficiency of model membranes in the presence of SNAREs

**DOI:** 10.1038/s41598-017-12348-w

**Published:** 2017-09-21

**Authors:** Torben-Tobias Kliesch, Jörn Dietz, Laura Turco, Partho Halder, Elena Polo, Marco Tarantola, Reinhard Jahn, Andreas Janshoff

**Affiliations:** 1 Institute of Physical Chemistry, Georg-August-University, Göttingen, 37077 Germany; 2 Max-Planck-Institute for Dynamics and Self-Organization, Göttingen, 37077 Germany; 3 Max-Planck-Institute for Biophysical Chemistry, Göttingen, 37077 Germany

## Abstract

The large gap in time scales between membrane fusion occurring in biological systems during neurotransmitter release and fusion observed between model membranes has provoked speculations over a large number of possible factors that might explain this discrepancy. One possible reason is an elevated lateral membrane tension present in the presynaptic membrane. We investigated the tension-dependency of fusion using model membranes equipped with a minimal fusion machinery consisting of syntaxin 1, synaptobrevin and SNAP 25. Two different strategies were realized; one based on supported bilayers and the other one employing sessile giant liposomes. In the first approach, isolated patches of planar bilayers derived from giant unilamellar vesicles containing syntaxin 1 and preassembled SNAP 25 (ΔN-complex) were deposited on a dilatable PDMS sheet. In a second approach, lateral membrane tension was controlled through the adhesion of intact giant unilamellar vesicles on a functionalized surface. In both approaches fusion efficiency increases considerably with lateral tension and we identified a threshold tension of 3.4 mN m^−1^, at which the number of fusion events is increased substantially.

## Introduction

Fusion of small membranous organelles inside a cell and fusion of vesicles with the plasma membrane are the key steps in the secretory pathway for the transport of lipids, proteins and signalling molecules in eukaryotic cells^1–3^. Neuronal exocytosis of synaptic vesicles involves many different proteins including SNAREs, Rab, RIM, Munc18, and synaptotagmins^4^. Docking and priming of synaptic vesicles at the presynaptic active zone membrane is mediated by at least five proteins (RIM, Munc13, RIM-BP, α-liprin and ELKS) that form a large complex connecting Ca^2+^ channels to the primed vesicles^5^. The control and guiding of synaptic vesicles towards fusion is essential for signal transduction from the presynaptic neuron to the postsynaptic cell. The minimal fusion machinery in the secretory pathway is composed of the soluble N-ethylmaleimide-sensitive-factor attachment receptor (SNARE) proteins that catalyse fusion. SNAREs assemble in a zipper-like fashion between two opposing membranes, resulting in a bundle of four intertwined alpha helices, which brings the outer leaflets into close contact^6–8^. It is well established that SNARE assembly provides the driving force for overcoming the energy barriers separating the intermediate states along the fusion pathway including stalk formation followed by the initial opening and the expansion of the fusion pore^8,9^. The heights of these energy barriers are rate limiting for fusion but it is still unclear how exactly they arise. While the lipid composition is clearly important, local influences are exerted by mechanical stresses such as curvature and lateral tension of the membrane^10^. The influence of curvature stress on fusion has been addressed in numerous studies^11,12^, however, much less is known how lateral membrane tension influences fusion kinetics. It is clear that lateral stress in the membrane exposes hydrophobic tails of the lipids and thereby reduces barriers associated with splaying of lipids and facilitates lipid insertion into the voids. Membrane tension is known to be involved in many biological processes such as membrane trafficking, cell shape, adhesion, growth and motility^13,14^. Tension in the plasma membrane originates mainly from attachment to the underlying actin cortex and osmotic pressure. In this context *Wen et al*. have recently studied the impact of F-actin connected to the plasma membrane of the cell on bilayer fusion. The finite membrane tension generated by the underlying F-actin resulted in full fusion of the vesicles with a depletion of the vesicles into the plasma membrane^15^. Also cell-cell fusion is induced by invasive protrusions generated by F-actin from an attacking cell. A mechanosensory response by the actomyosin network of the receiving cell also provides tension in the plasma membrane as a resisting force to the invading cell membranes^16^. The fact that membrane tension is generated by the connected, underlying cytoskeleton network of F-actin supports the assumption that membrane tension can be locally increased at defined membrane areas (e.g., active zones) to guide vesicle fusion by increasing hydrophobicity at the contact zone.

*Shillcock* and *Lipowsky* postulated that fusion of bilayer membranes with vesicles is tension-induced and they performed molecular dynamics (MD) simulations that clearly show how an increase in lateral tension facilitates fusion^17^. Their simulations showed that a tensionless vesicle only adheres to the lipid bilayer, while tension exerted in both of the opposing bilayers led to a higher rate of full membrane fusion events. Also, alternative pathways arise, for instance, tensed membranes may rupture at elevated tension, while at lower tension, the hemi-fused state can expand, thereby relaxing membrane tension by providing excess membrane area. It is clear that the probability of successful fusion rises with tension but might run through an optimum since larger tension might inevitably lead to uncontrolled membranes rupture, while fusion probability decreases for smaller tensions as adhesion of the vesicle and hemifusion become more favourable. It is therefore suggested that the tension has to exceed a certain threshold value in order to induce fusion but might also level off at larger stress. Along these lines *Kozlov* and *Chernomordik* suggested that a more sophisticated analysis of membrane tension is necessary to address the impact of tension in fusion assays for a better understanding of biological fusion processes^18^.

In spite of all these indications, the hypothesis that elevated membrane tension increases vesicle fusion efficiency has not yet been verified experimentally. In order to do this, one needs to change the available membrane area within a small range from 0–5% of its initial area. Here, we generated global tension in lipid bilayer either by dilatation of supported bilayers or stretching adhered giant liposomes by adjusting the adhesion with the substrate. Lateral stretching of supported lipid bilayers was achieved using a millifluidic device, which permits us to address the full range from slightly negative to lysis tension with high accuracy. Tension is adjusted by applying a vacuum to adjacent channels of this device and the membrane area change of membrane patches is measured using an optical microscope in conjunction with the thresholding technique of *Li et al* ^19^. Stretching and compression of a lipid bilayers on an elastic PDMS sheet was first established by *Staykova et al*. using changing air pressure in a chamber with a thin PDMS layer on top^20^. We were particularly inspired by the work of *Huh et at*. who investigated the mechanical properties of cells on stretchable substrates to mimic the biological environment in an organ that is exposed to mechanical deformation^21,22^. Replica modelling of these constructions provided us with a device bearing a thin PDMS sheet spanning between two side channels, where air pressure could be reduced to achieve an increased surface area on the sheet. This in turn can be used to increase the area of adhered lipid bilayers. Alternatively, we also used adhered giant unilamellar vesicles (GUVs), where the lateral membrane tension was adjusted through the adhesion of the GUVs with a functionalized glass surface. The adhesion area and thus lateral membrane tension of the GUV was increased through an elevated concentration of Mg^2+^-ions in the buffer leading to stronger attractive forces between the bilayer and the substrate^23^. The vesicle fusion efficiency as a function of the corresponding tension adjusted in the supported lipid bilayer (SLB) and GUV-membrane was monitored by fluorescence microscopy. We equipped our bilayers with a minimal fusion system consisting of SNAREs comprising synaptobrevin (1–116), syntaxin1A (183–288), and SNAP-25 (1–206)) to obtain reasonable fusion activity. With these experimental setups, we could monitor the area change of each membrane patch after stretching the substrate and document the fusion of LUVs added to the solution. These systems allow for the first time to quantitatively investigate the impact of membrane tension on vesicle fusion. We found that the fusion efficiency of large unilamellar vesicles (LUVs) with SLBs was highly increased above a threshold tension corresponding to a membrane area dilation of approximately 1.2%. Larger tension shows only slightly altered fusion efficiency.

## Results

**Figure 1:**
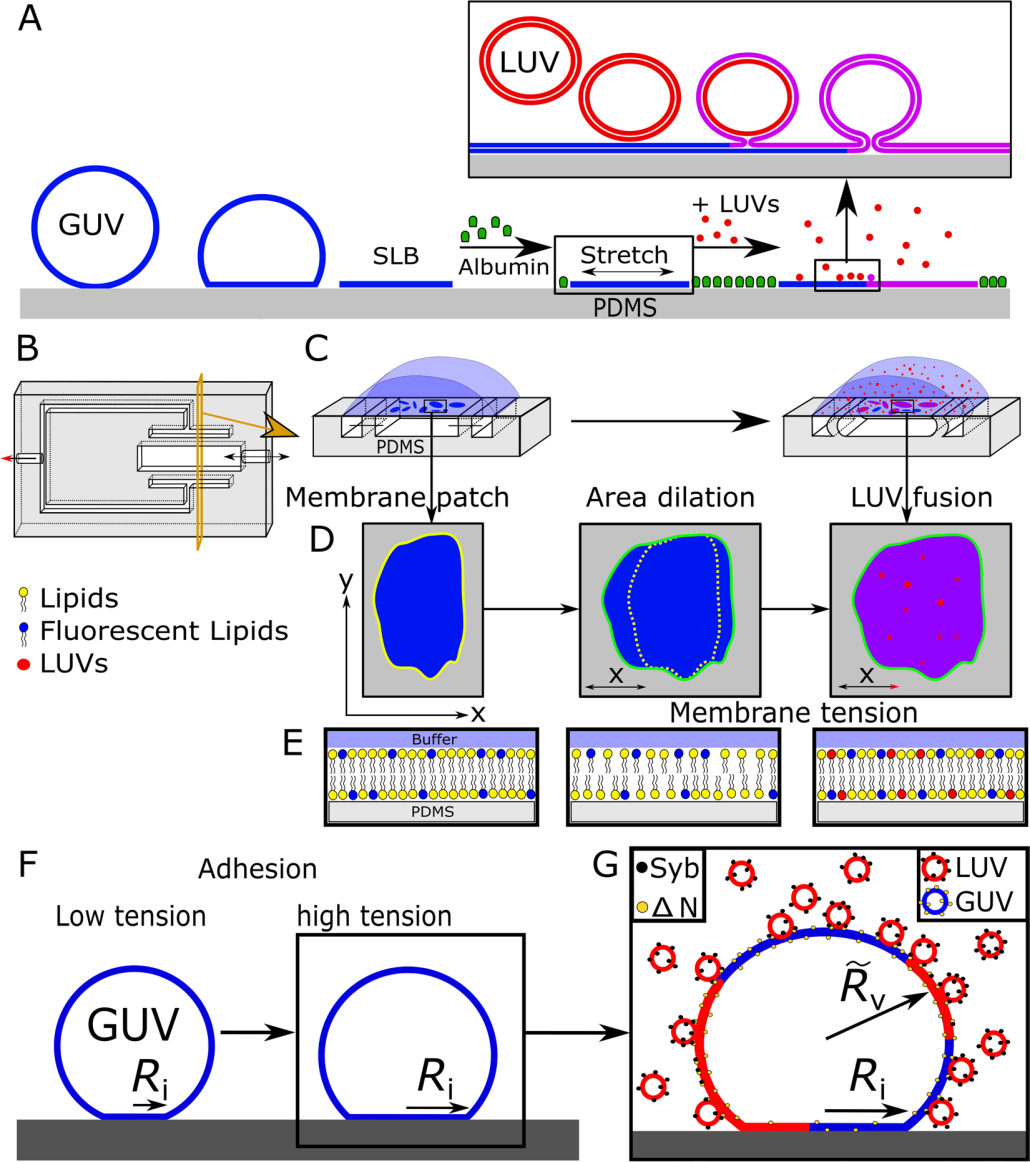
Scheme of the two assays to measure membrane fusion as a function of lateral tension. (**A**) Illustration of the experiment to measure tension-dependent fusion of LUVs (red) to pre-stretched bilayers (blue) based on dilatable PDMS sheets. GUVs (blue) were incubated with the hydrophilic PDMS surface in HEPES-buffer and forced to spread forming membrane patches of defined size. Albumin buffer solution was used to passivate the surface inhibiting LUV fusion on the neat PDMS. The addition of LUVs to the sample resulted in fusion of LUVs with the SLBs in presence of SNARE proteins. (**B**) Construction of the PDMS chamber: a large central chamber with a connection to the environment has two adjacent side channels. When the pressure in the side channels is reduced, the thin PDMS sheet on top will dilate. (**C**) Cross sectional views of the three-chamber PDMS device illustrating the sequence of events leading to tensed SLBs and eventually fusion with added LUVs. (**D**) Stretching of the underlying PDMS sheet could induce an area increase in between 0 to 5% of the initial area of the bilayer patches. Docking, hemi-fusion, and full-fusion of LUVs to the SLBs were monitored with fluorescence microscopy. (**E**) Stretching of the lipid bilayer leads to an increase in hydrophobicity (cross-section) fostering fusion. (**F**) Generation of pre-stressed GUVs by modifying adhesion to the functionalized glass surface. (**G**) SNARE mediated fusion of LUVs to adhered GUVs could be measured by lipid and content mixing. The GUV-membrane tension was calculated by evaluating the shape of the sessile GUV, i.e., the radius of the vesicle (${\tilde{R}}_{{\rm{V}}}$) and the contact radius (*R*_i_) with the substrate.

Lateral membrane tension describes the stresses of a lipid bilayer at every point along the surface and the surrounding medium, which is the cytosol in cells^18^. Three main sources are responsible for mechanical tension in lipid bilayers: (i) osmotic pressure between the cytosol and the surrounding medium of the cell or vesicle;^24,25^ (ii) interaction of the cytoskeleton or with the plasma membrane;^15^ and (iii) adhesion forces to surfaces or other cells^18,23,26^. In order to simulate tension in artificial systems one could in principle realise an osmotic imbalance, employ a defined adhesion strength to laterally dilate sessile liposomes or apply lateral stress directly to a supported bilayer. Here, we use the two latter strategies to cover a broad range of tension values ranging from stress-free bilayers to lysis tension where cracks start to appear in the membrane. Figure 1 illustrates the general idea how we envision to generate tension in supported membranes and adhered liposomes and how we monitor SNARE-assisted membrane fusion of LUVs with these bilayers. First, a stretchable PDMS-sheet covered with unconnected, isolated membrane patches derived from lysed GUVs was used to exert a defined lateral tension by area dilatation. Confined membrane patches that do not exchange lipids with adjacent membranes are necassary since stretching is found to be anisotropic over the surface and the precise area dilatation would not be accessible for a continuous bilayer. As a dilatable substrate, a freestanding PDMS sheet with a thickness of approximately 180 µm was used (Supplementary Figure S1). The PDMS sheet was attached to two adjacent channels as shown in Fig. 1B,C. Lowering air pressure with a syringe pump attached to the side channels resulted in a mild expansion of the PDMS surface and in turn also in dilatation of the attached lipid bilayers (Supplementary Figure S2). We found that the adhesive strength between the membrane and the PDMS is sufficiently strong to keep the bilayer coupled to the PDMS sheet even if it is stretched. This was not unexpected since the adhesion energy together with osmotic stress used to rupture the GUVs was initially sufficient to overcome the lysis tension of GUVs. Naturally, the extension of the PDMS surface is anisotropic. In the middle of the sheet between the side chamber walls the area dilatation is close to zero but increases towards the side wall, as previously visualized by *Huh et al*. using quantum dots sticking to the PDMS surface^22^. Here, in our experiments the anisotropy is an advantage since it allowed us to generate a broad spectrum of different membrane area changes within one sample preparation. How do we obtain the tension from the area change? The overall isotropic in-plane tension σ of a lipid bilayer is a function of the pretension σ_0_, which results mainly from adhesion stress or initial osmotic differences, the area compressibility modulus *K*_A_, and the area change Δ*A*/*A*_0_ of the lipid bilayer. ΔA = *A*_m_−*A*_0_ denotes the difference between the actual membrane area *A*_m_ and the initial membrane area prior to extension *A*_0_^27^:

1 $${\rm{\sigma }}={\sigma }_{0}+{{K}}_{{\rm{A}}}\frac{{\rm{\Delta }}A}{{A}_{0}}$$

Equation (1) neglects the presence of thermally excited membrane undulations and is only applicable for large membrane tension where membrane undulations are suppressed. While equation (1) captures the case of laterally dilated supported bilayers well, undulations need to be taken into account for weakly adhered vesicles. Therefore, we employ equation (2) to relate the membrane area change of a vesicle membrane to the lateral tension of the bilayer. As previously described we assume a pre-stress tension of *σ*_0_ = 9.7 × 10^−5^ N/m for vesicles prior to adhesion:^23,28,29^

2 $$\frac{{\rm{\Delta }}A}{{A}_{0}}=\frac{\sigma -{\sigma }_{0}}{{K}_{{\rm{A}}}}+\frac{{k}_{{\rm{B}}}T}{8\pi \kappa }\,{\rm{l}}{\rm{n}}(\frac{\sigma }{{\sigma }_{0}})$$

The first term on the right-hand side describes the area dilatation according to Hooke’s law as described before and the second term contains the excess area stored in the thermal undulations of the membrane. Here, we assume a bending rigidity κ = 0.85 × 10^−19^ J ≈ 21 *k*_B_ *T* ^30^. With equation (2) the membrane tension *σ* of adhered GUVs was calculated numerically.

### PDMS-based fusion assay

The membrane tension of SLB patches on the PDMS surface depends linearly on the relative change in membrane area. From equation (1) we know that the absolute tension in the bilayer can be computed for a reference pre-stress *σ*_0._ Since *σ*_0_ is not precisely known for each membrane patch we rather refer to the tension difference *σ*-*σ*_0_ as the lateral tension. Notably, adhesion to the substrate renders the freshly deposited membrane not tension-free. It is, however, generally possible to compensate for this initial stress by relaxing the system using higher air pressure in the channels. We assume an area compressibility modulus of *K*_A_ = 0.28 N m^−1^ for this lipid composition as determined previously^28^.

We used a minimal SNARE machinery with Syb reconstituted in the LUVs and the ΔN-complex displayed by the SLBs or GUVs^31,32^. In previous studies we and others could show that this lipid-protein composition is sufficient to accomplish merging of membranes within seconds^33,34^.

**Figure 2:**
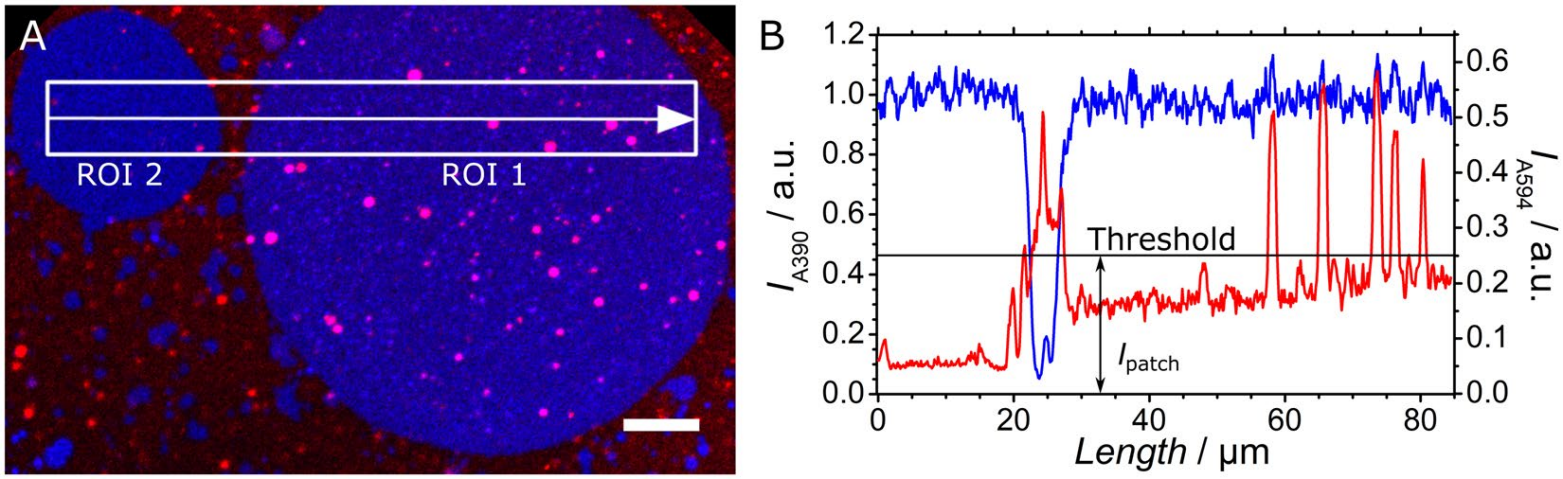
Fluorescence microscopy images of membrane patches illustrating docking, hemi-fusion and full fusion with LUVs. (**A**) Fluorescence image (red and blue channel overlay) showing two membrane patches (ROI 1 and 2) after applying lateral stress together with a white box at which the intensities of dye A390 and A594 are averaged and plotted in (**B**). (**B**) The area scan shows the mean intensity of both dyes taken from the white box shown in (**A**) using the corresponding color for each dye. Both intensities were normalized by the mean A390 emission of the SLB patches as a reference. The lipid dye A594, initially only present in the LUVs, emits less light from ROI 2 with a tension of (1.1 ± 0.8) mN m^−1^ compared to ROI 1 that reached a membrane tension of (4.4 ± 0.8) mN m^−1^. A threshold for the mean intensity *I*_patch_ was chosen (black line) to measure the fusion efficiency *F*_eff_ (Eq. 3) by lipid mixing so that higher intensities from docked LUVs were excluded. Scale bar: 10 µm.

**Figure 3:**
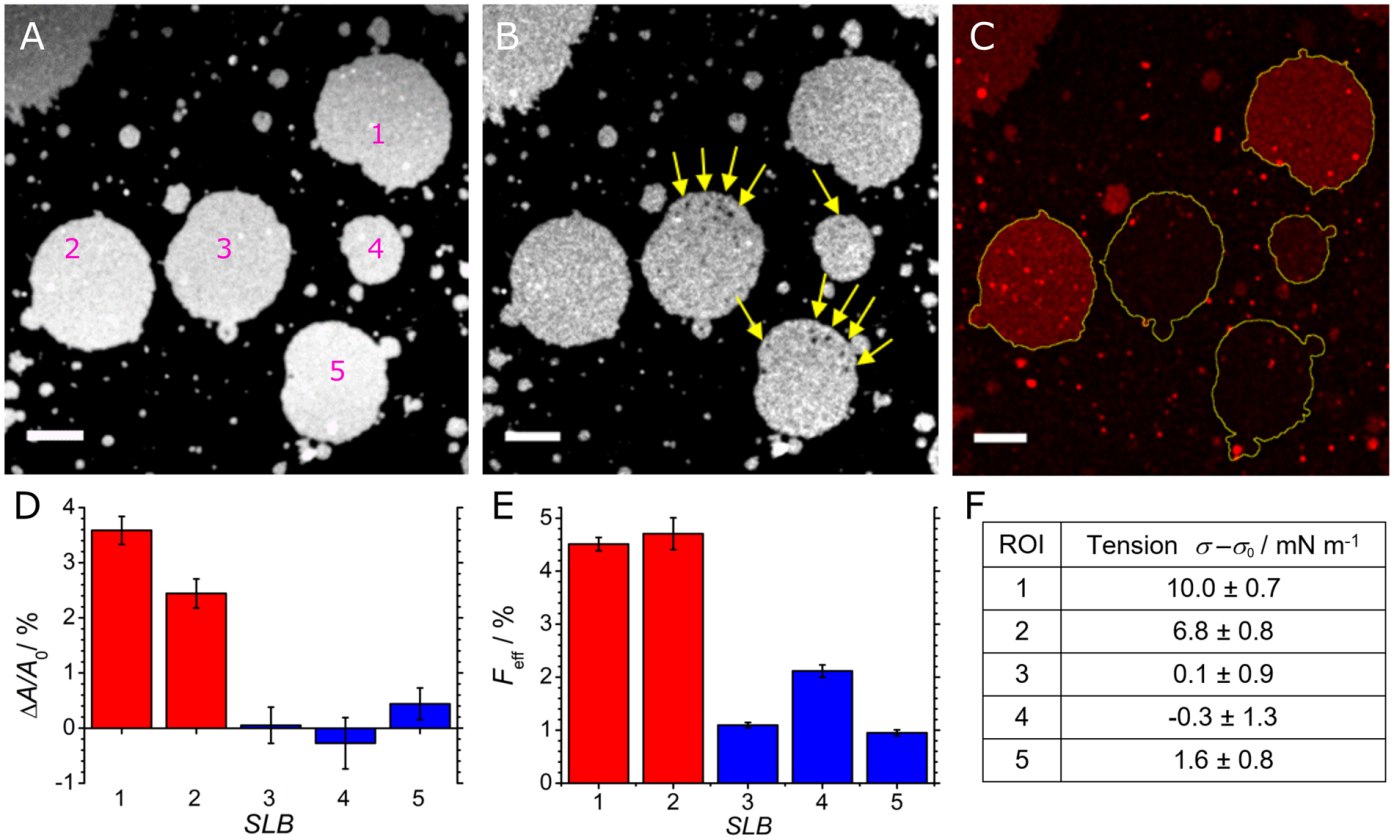
Membrane fusion of LUVs (red) to pre-stressed SLBs (white) monitored with confocal microscopy. (**A**) SLBs with an individually defined membrane area were created from spreading of GUVs containing the blue dye A390 on an expandable PDMS surface. (**B**) After stretching the patches, the membrane area of some of the patches increases. Frequently, holes in the bilayer may also appear (SLBs 3, 5) as highlighted by yellow arrows. (**C**) Fluorescence image of the same position showing emission of the red dye A594 after addition of LUVs. Fusion of LUVs was only observed with stretched planar membranes (SLBs 1, 2). Scale bar: 10 µm. (**D**) Relative membrane area change of the GUV patches after expansion of the PDMS sheet. SLBs 1, 2 show a substantial membrane area increase due to stretching of the PDMS sheet, while SLBs 3, 4, and 5 remain almost unstressed. (**E**) Fusion efficiency (Eq. 3) of LUVs at the different SLBs. SLBs 1, 2 show a significantly higher fusion efficiency compared to SLBs 3, 4, and 5. (**F**) Tension values obtained from Eq. (1) using the corresponding area dilatation.

The experiment to monitor fusion as a function of tension on PDMS-based lipid bilayers comprises three stages. First, the PDMS sheet was covered with GUVs that, assisted by an osmotic imbalance, immediately spread upon contact to form isolated membrane patches on the PDMS. The size of these patches labelled with the lipid dye A390 was measured by thresholding as described in the experimental part (*vide infra*). Second, the PDMS with the attached SLBs was dilated until a defined extension was reached and kept in this tensed state for the remainder of the experiment. Again, the increase in membrane area was documented by imaging the fluorescence emitted by the lipid dye A390. Using equation (1) the area increase was used to compute the lateral tension of the patches depending on their location on the PDMS sheet. Third, the stretched membrane patches were incubated with LUVs labelled with the red dye A594. Images were taken consecutively to monitor docking and (hemi)fusion events taking place on the dilated membrane patches. In order to avoid that the emission of A594 from docked and hemifused vesicle is falsely subsumed as post fusion - fluorescence emitted from the membrane patch we used a threshold that excluded docked and hemifused vesicle from being counted (see Fig. 2). Fusion of vesicles to the edges of membrane patches was prevented by passivation of the hydrophilic PDMS surface with an albumin buffer solution (Fig. 1, see also Supplementary Figures S3 and S4). Fusion of vesicles to the edge of the membrane patches was absent in all experiments where the PDMS surface was passivated with albumin. Lipid mixing and docking of vesicles was quantified by measuring the relative fluorescence intensity *I*_*patch*,_ of the dye A594 originating from the LUVs to the internal reference, i.e., the maximum intensity *I*_max_ of the dye A594 in the image which is found by to be emitted from adhered LUVs on the PDMS surface (Supplementary Figures S5–S8). In essence, fusion efficiency was defined as

3 $${F}_{{\rm{eff}}}=\frac{{I}_{{\rm{patch}}}-{I}_{{\rm{back}}}}{{I}_{{\rm{\max }}}-{I}_{{\rm{back}}}}\cdot 100 \% ,$$

with *I*_back_ the background fluorescence intensity emitted from the dark area surrounding the membrane patch. Note that this procedure renders efficiencies rather low since fusion relaxes tension and naturally limits the amount of fused vesicles. The method to use the fluorescence intensity of the lipid dye from the incubated LUVs as a reporter for fusion (lipid mixing) was validated with conventional vesicle fusion assays and turned out to be in good agreement with previous work^31,35^. In Fig. 3, five membrane patches are shown in the relaxed state prior to applying a vacuum to the adjacent channels (A), after stretching of the substrate (B), and after incubation with LUVs (C). SLBs and LUVs were equipped with the SNARE fusion machinery as described in the Materials and Methods section. Relaxed and stretched membrane patches showed a constant area over time, as measured by fluorescence microscopy employing the outlined thresholding procedure^19^. This is an important prerequisite for a trustworthy analysis of subsequent fusion events. Stretching of the PDMS substrate led to a noticeable increase in membrane area (SLBs 1, 2). However, some membranes did not show a detectable area change (SLBs 3–5) albeit being stretched. We attribute this to rupture events within the membrane patch, which create small holes in the bilayer that relax tension. As a consequence, the area formed by the holes was subtracted from the overall area of the patch and thereby excluded from area determination (SLBs 3, 5). Therefore, albeit the boundary of the patch grows, the area sometimes remains constant due to occurrence of holes in the bilayer. After addition of fusogenic LUVs equipped with SNARE proteins we found a considerably increased mean fluorescence intensity of the LUV dye (Fig. 3C+E) within SLBs 1, 2 representing dilated membrane patches (Fig. 3D). We attribute this to lipid mixing of SLB lipids with lipids from the added LUVs diffusing into the stretched patches. In contrast, the three other membrane regions (SLBs 3–5), which only display a small area change upon stretching the PDMS sheet, show only a small increase in relative fluorescence intensity of the LUV dye A594. Along the same lines, Fig. 2 (see also Supporting Figure S8C+D) shows exemplarily two adjacent membrane patches (ROI 1, 2), in which one remained unstressed (ROI 2) after dilatation of the PDMS matrix and the other one assumed an expanded state (ROI 1). The SLB of ROI 1 increased its area even after subtraction of the non-fluorescent area formed by the holes. LUV fusion occurs only on ROI 1 showing a relative area increase of (1.6 ± 0.3) % corresponding to a lateral membrane tension of (4.4 ± 0.8) mN m^−1^ (Supplementary Figure S8E). In contrast, ROI 2, which experienced only an area increase of (0.4 ± 0.3) % translating in a lateral tension of (1.1 ± 0.8) mN m^−1^, does not show a significant number of fusion events.

In general, we did not observe a noticeable increase in area of the membrane patch after fusion. We therefore conclude that relaxation of lateral tension in the membrane by supply of lipids from LUV fusion arrests if the excess area generated by stretching of the bilayer is exhausted (Fig. 2 ROI 1). After the lateral tension is fully relaxed by refilling the voids with external lipids from fusing with LUVs, arrested (hemi)fusion and docking of intact LUVs remain the only option to interact with the membrane patch. The line scan through both membranes displayed in Fig. 2B shows a higher mean fluorescence intensity at ROI 1 together with peaks originating mainly from adsorbed LUVs. Lipid bilayer fusion between LUVs and the SLBs was detected at the points where peak fluorescence intensities overlapped for both channels (Fig. 2 B). Since full lipid insertion no longer occurs due to the inability of the SLB to expand laterally, the fused LUV stays arrested on top of the SLB but shares lipids with the SLB. Docked vesicles are only found on ROI 2 displaying no fluorescence emitted from dye A390 indicating that lipid mixing did not occur. In comparison, eight LUVs docked to the supported membrane at ROI 2 compared to more than 30 docked LUVs on the membrane at ROI 1. A total number of 36 fused LUVs could be detected at ROI 1 compared to none at ROI 2. Control experiments in which both SLBs and LUVs lack SNARE proteins entirely or partly showed no significant fusion efficiency (Supplementary Figure S4). Therefore, we conclude that membrane fusion of LUVs in the presence of SNAREs occurs more frequently with dilated membrane patches compared to stress-free membranes.

FRAP experiments carried out on membrane patches after fusion of LUVs clearly show that lipids originating from LUVs are inserted in a fully mobile fashion into the patch (Supplementary Figures S9–S12). Diffusion constants of the lipid dye A549 are found to be around 0.6 µm^2^/s with an immobile fraction of 15% independent of lateral tension. Additional FRAP experiments performed on LUVs attached to the membrane patch targeting to bleach A594 confirmed that lipids are shared between the SLB and the LUVs. We can therefore assume that some vesicles are arrested in a stable hemifusion intermediate and do not fuse with the membrane patch, which we attribute to lack of free volume within the bilayer patch^35^. The tensed SLB (ROI 1) in Fig. 2 displays a considerable number of docked and hemi-fused vesicles compared to the unstressed SLB of ROI 2. We attribute the increased affinity of vesicles to stretched membrane patches to the enhanced hydrophobicity of the expanded SLB fostering sticking.

**Figure 4:**
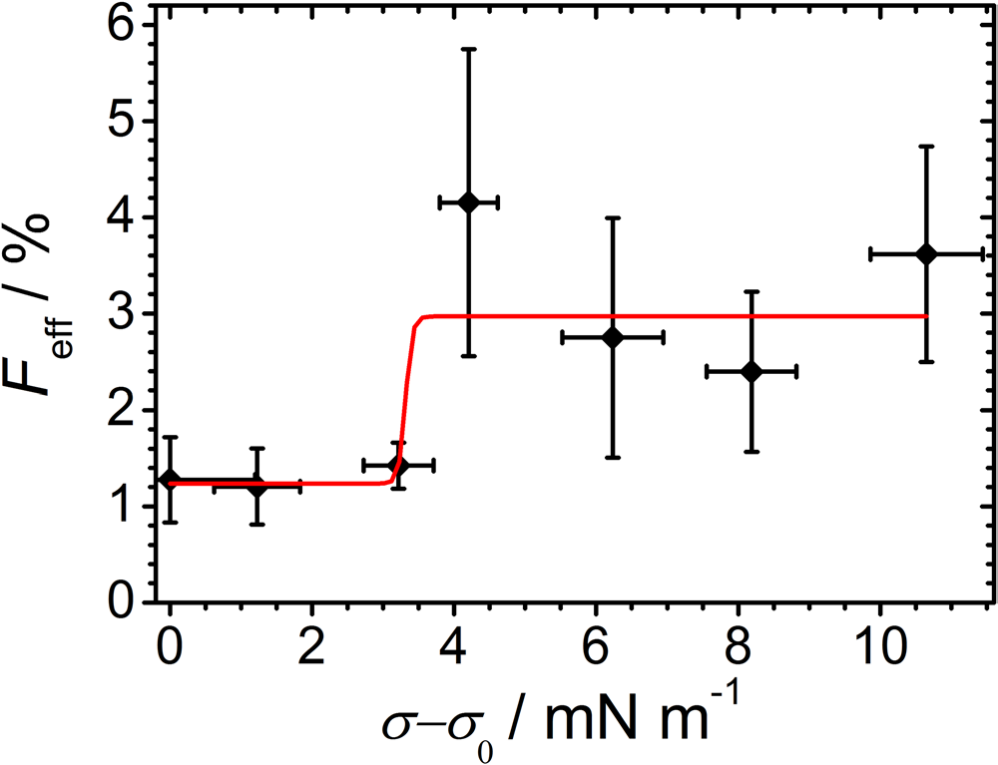
Fusion efficiency as a function of lateral membrane tension of supported lipid bilayers. Membrane fusion efficiency of LUVs to SLBs as a function of lateral membrane tension (black dots, red line is a Hill fit to guide the eye, *n* = 30 membrane patches were pooled). Tension was computed from area expansion according to Eq. 1 assuming an area compressibility modulus *K*_A_ of 0.28 Nm^−1^. Fusion efficiency is defined in Eq. 3.

Figure 4 shows how fusion efficiency depends on lateral tension of the SLB deposited on the dilatable PDMS-sheet. The results show that membranes pre-stressed to a tension up to 3.2 mN m^−1^ display an only moderate increase in fusion efficiency translating in a fusion efficiency of (1.3 ± 0.4)%. Above a membrane tension of 4 mN m^−1^, a significant increase in relative fluorescence intensity originating from fused LUVs was measured. At around 1.4–1.6% area dilatation corresponding to a membrane tension of 4.0–4.4 mN m^−1^, the relative fluorescence intensity increases up to (4.2 ± 0.4)%. A further elevated membrane tension above 5 mN m^−1^ does not result in higher fusion efficiencies but stays constant at a level of around (3.2 ± 1.2)%. Therefore, we state that a threshold tension is necessary to be conquered in order to boost fusion. Since the number of fused vesicles is limited by the area difference between the stretched patch and the one in the relaxed state the fusion efficiency is restricted to a few percent only. In order to remove some of the drawbacks associated with supported bilayer such as lack of constant tension during fusion we also used pre-stressed GUVs to monitor fusion as a function of tension (*vide infra*).

### GUV-based fusion assay

A natural drawback of using PDMS membranes is the presence of a solid support that may interfere with essential steps in successful fusion and also alters the energy landscape since adhesion energy needs to be spend in order to peel of the membrane^35^. Therefore, we also employed an assay based on sessile GUVs with adjustable tension. As described previously, it is feasible to control the adhesion area of the GUV in contact with the substrate and thus adjust the lateral membrane tension of the GUVs in a broad range^23^. This was achieved by controlling the concentration of Mg^2+^-ions in solution. In general, GUVs were attached to the substrate using an avidin-coated silicon surface and biotinylated phospholipids reconstituted in the GUV. The negative charge of the GUV surface can be used to carefully adjust the size of the contact zone by changing the concentration of Mg^2+^. Due to the bridging effect of Mg^2+^ adhesion increases with increasing Mg^2+^ concentration. Consequently, the membrane tension of the GUVs could be adjusted between 0.1 mN/m and 5 mN/m. In the limit of strong adhesion tension is obtained from the geometry of the adherent GUV since the GUV assumes the shape of a sessile drop. A three dimensional image stack of the GUVs revealed the actual vesicle radius and adhesion radius, which is sufficient information to compute the membrane tension^23^. Fusion with LUVs can readily be quantified by collecting fluorescence emission from the lipid dyes (A594 or A488) of the contact zone. This procedure allows to exclude adhered vesicles from the analysis and to use FRAP in order to confirm that the lipids are freely mobile in the GUV.

**Figure 5:**
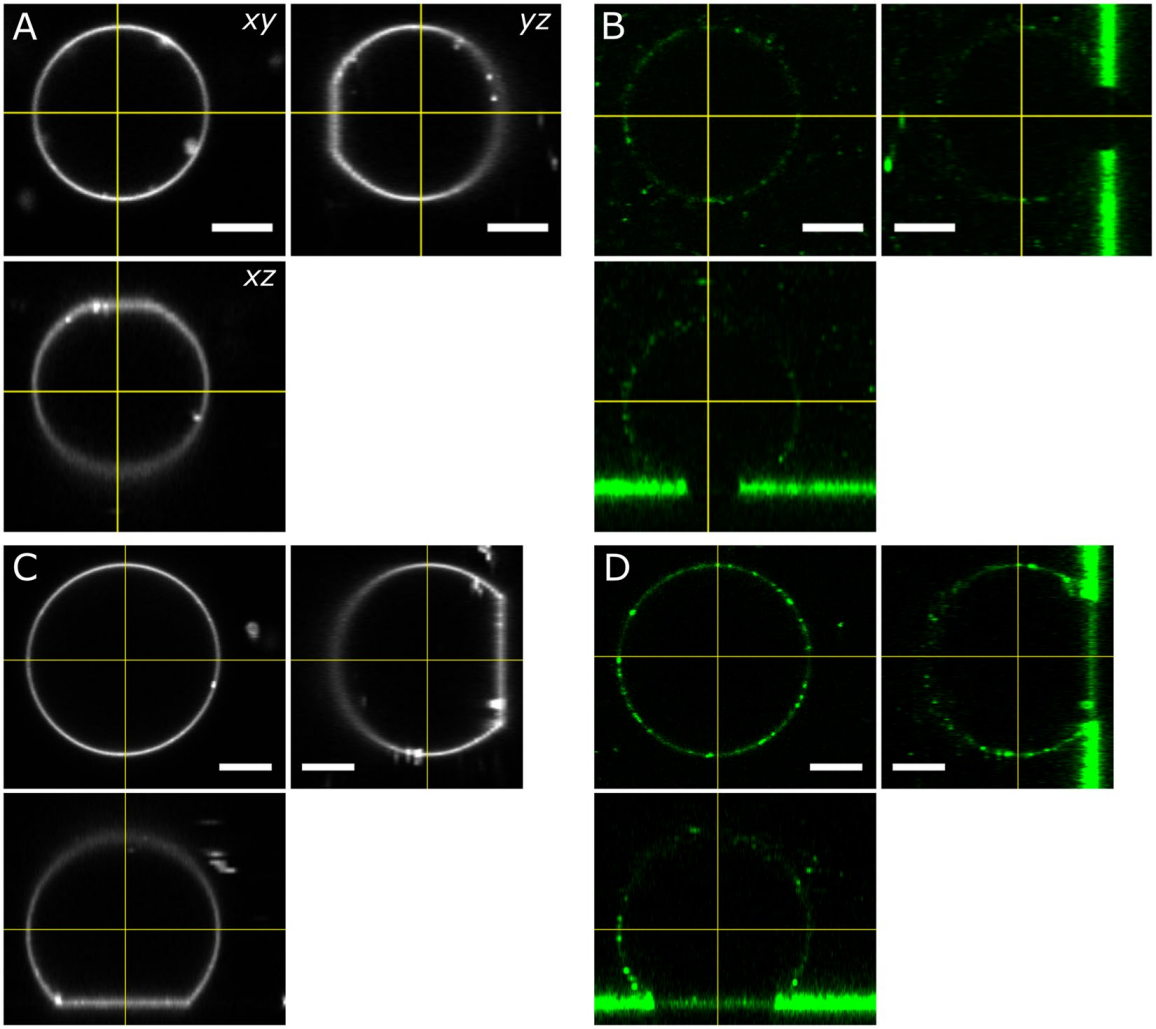
LUV fusion to pre-stressed GUVs. (**A**) Confocal laser scanning micrographs of sessile GUVs bound to the surface via specific (biotin-avidin) and non-specific interactions (Mg^2+^). A small adhesion area indicates low membrane tension of (0.30 ± 0.03) mN/m. (**B**) The amount of docked LUVs (green) to the GUV-membrane is low and lipid mixing could not be detected (no fluorescence in the contact zone). (**C**) GUV with a large adhesion area corresponding to a high membrane tension of (4.0 ± 0.5) mN/m. (**D**) The amount of docked LUVs (green) to the freestanding part of the GUV-membrane is much higher compared to lower membrane tensions. Lipid mixing was confirmed by verifying that the green dye from the LUV (A488) is present also in the adhesion zone, where docking of vesicles is impossible. The bright fluorescence intensity on the substrate surface around the GUV-adhesion area is emitted from adhered LUVs that settled on the surface. The fluorescence intensity of the LUV dye in (**D**) at the adhesion site of the tensed GUV is substantially higher compared with the less tensed GUV shown in (**B**). Scale bar: 5 µm.

Figure 5 shows exemplarily two GUVs, one with a small adhesion area bearing a low membrane tension of (0.3 ± 0.03) mN/m (Fig. 5 A) and one with a large adhesion area corresponding to elevated membrane tension of (4.0 ± 0.5) mN/m (Fig. 5 C). On the right side of Fig. 5, the fluorescence emitted from the LUV-membranes indicates that only a small amount of LUVs dock to the unstressed GUV-membrane (Fig. 5B, see Supplementary Figures S13–S15). Conversely, at a higher membrane tension the number of docked LUVs is largely increased (see Supplementary Figures S16–S19) and the adhesion zone of the GUV-membrane shows a strong fluorescence emission from the LUV dye, which indicates that LUVs fused with the GUV membranes. Membrane exhibiting tensions below 1.2 mN/m show only a small number of docked LUVs and nearly no lipid mixing (see Supplementary Figures S13–S15). In general, an increased fluorescence intensity from the adhesion zone could only be observed for strongly adhered GUVs bearing a higher membrane tension (see, for example Fig. 5D).

FRAP measurements performed at the contact zone targeting the LUV dye A488 or A594 confirm that lipid mixing between LUVs and the adhered GUVs occurred (Supplementary Figures S20–S22). A content mixing assay where LUVs were filled with a water-soluble dye showed that LUVs fully fused with the tensed membrane of the GUV 28 (see Supplementary Figures S23–S25). A partial or full lack of SNARE proteins in the GUVs and LUVs does not lead to any LUV fusion even for highly tensed GUV membranes (see Supplementary Figures S26).

### Comparison of both fusion assays

Figure 4 shows the fusion efficiency between LUVs and pre-stressed planar bilayers as a function of lateral membrane tension. Fusion efficiency was calculated from Eq. 3 in which *I*_patch_ was substituted by the intensity emitted from the contact zone of GUV with the substrate *I*_adhesion_. For all 30 membrane patches, the membrane area changes and relative fluorescence intensities were measured from two up to 10 images 30 min after LUV incubation and subsequent rinsing of the substrate. The average error in determining the relative change in area was usually in the range of Δ|ΔA/A_0_| = (0.4 ± 0.2)%. Since smaller patches are more prone to erroneous determination of area change only membrane patches with a size range from 100 µm^2^ to 7000 µm^2^ were used for analysis of patch area and fusion efficiency ensuring that the overall area change error was below 1%. The main sources of error are photobleaching of the oxidation-sensitive lipid dye A390 and small changes of the focal plane.

**Figure 6:**
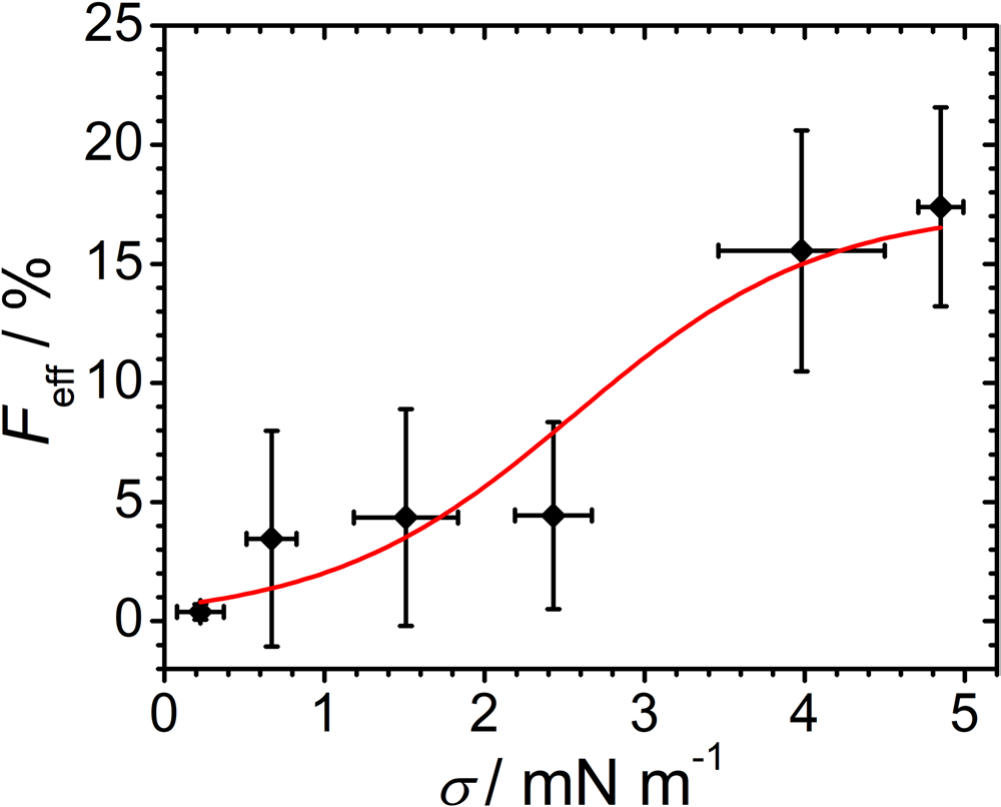
Fusion efficiency as a function of membrane tension in GUVs. Fusion efficiency of LUVs to adhered GUVs is plotted as a function of lateral GUV-membrane tension (black dots, red line is a Hill fit to guide the eye, *n* = 19 GUVs were pooled). Tension was computed from area expansion according Eq. 2 for assuming an area compressibility modulus *K*_A_ of 0.28 Nm^−1^, a bending rigidity *κ* of 21 *k*_B_ *T* and a pre-stress-tension *σ*_0_ of 9.7 × 10^−5^ Nm^−1^. Fusion efficiency is defined in Eq. 3.

Figure 6 shows the fusion efficiency between LUVs and adhered GUVs as a function of lateral membrane tension. For slightly adhered GUVs with low membrane tension ranging from zero to 0.44 mN/m, the fusion efficiency is very small (0.4 ± 0.3)%. An elevated GUV-membrane tension between 0.6 mN/m to 2.6 mN/m increases the fusion efficiency slightly to (4.0 ± 4.0)%, whereas at GUV-membrane tensions between 4 mN/m and 5 mN/m the fusion efficiency increases drastically to (16.5 ± 4.6)%. The generally much higher fusion efficiencies found for adhered GUVs compared to PDMS-based supported lipid bilayers under pre-stress are presumably due to a much larger tension reservoir in the case of adhered GUVs. While in PDMS-based membrane patches tension is relaxed after fusion, sessile GUVs grow and maintain the tension so that fusion lasts until the number of ΔN-complex copies in the GUV are exhausted. FRAP measurements carried out at the adhesion site of GUVs revealed that LUVs fuse and show lipid mixing with the adhered GUVs at higher membrane tensions (see Supplementary Figures S21 and S22). Both fusion assays showed an increased fusion efficiency at higher membrane tension. Notably, the transition to higher fusion efficiencies for the adhered GUVs is in the same tension regime (4–5 mN/m) as for the dilated SLBs but the effect is more pronounced in the former case.

In summary, we found that fusion efficiency increases considerably once a threshold membrane area dilatation of 1.2% corresponding to a membrane tension of 3.4 mN/m is reached. Higher tension values than 5 mN/m do not significantly enhance fusion further. Therefore, we propose that the optimal tension range lies between 3.4–5 mN/m to considerably increase fusion efficiency compared to unstressed membranes.

## Discussion

The key finding of our study is that the probability of membrane fusion increases when external tension is applied to one of the participating membranes. Here, we gradually increased tension by area expansion of supported membranes and adhered giant vesicles below the lysis tension of the bilayer. Supported lipid bilayers were obtained from spreading of GUVs on a dilatable PDMS membrane. This ensures accurate access to the area of an isolated membrane patch. Stretching of the underlying PDMS sheet was achieved by using a millifluidic chamber with adjacent pressure channels that could precisely dilate the PDMS sheet and with it also the attached membrane patches with an accuracy of approximately 0.5% for an area of 900 µm^2^.

Interestingly, we detected membrane sliding, rupture and area increase in the same sample when stretching the PDMS support (Fig. 3). Membrane sliding means that the area of the membrane patch stays nearly constant, while stretching the underlying PDMS support. In essence, in this case the adhesion forces are not sufficiently strong to keep up with the traction forces. Besides, holes frequently appear in strongly adhered membrane patches in response to stretching of the underlying substrate and as a consequence, the membrane area stays nearly constant compared to its initial area in the relaxed state. Finally, the most frequent and relevant scenario is that the membrane follows the expansion of the PDMS substrate.

Adhered giant unilamellar vesicles with a defined adhesion area were obtained by incubating them on an avidin-functionalized glass surface. The Mg^2+^-ion concentration mediated the interaction of the biotinylated headgroups of the lipids in the GUV membrane with the avidin on the substrate surface. At higher Mg^2+^-ion concentrations the adhesion area of the GUVs grew and in turn also the membrane tension.

A minimal fusion machinery consisting of syntaxin, SNAP 25 and synaptobrevin as described previously was employed to obtain a reasonable fusion efficiency to begin with^33,34^. We also used lipids with the headgroup phosphatidylethanolamine that are known to result in negative spontaneous curvature in membranes and thus promote fusion processes. In regulated exocytosis of secretory vesicles cholesterol is concentrated in the membrane and has a major impact on vesicle fusion by clustering of the SNAREs and the formation and stability of fusion pores. Without cholesterol, most vesicles become arrested at hemi-fusion whereas an increased amount of cholesterol (30–40 mol%) results in full fusion of vesicles^36^. In our measurements we used 11 mol% of cholesterol and found mostly full fusion of vesicles to the tensed SLBs but also some hemi-fusion vesicles if the area expansion of the SLB is exhausted.

In the partial or full absence of fusogenic proteins, lipid bilayers do not show a significant number of fusion events in the experimental time frame of around 30 min regardless of the applied tension and assay. We found that unstressed membranes equipped with SNARE proteins show only few fusion events, while a membrane tension above 4–5 mN/m corresponding to a relative area increase of 1.4–1.8% increases fusion efficiency substantially. In essence, we found maximal fusion efficiency *F*_eff_ for a membrane tension in the range of 4–10 mN/m regardless whether GUVs or SLBs were used as host membranes. This observation is in good accordance with a theoretical study of *Grafmüller et al*. using dissipative particle simulations^37^. The authors found that fusion probability is maximal at intermediate tensions. While membrane rupture limits fusion probability at very large tensions, low tensions do not lower the energy barrier of fusion sufficiently since a relaxed planar bilayer has not enough space for lipids pouring in from LUVs. At low tension, either the hemi-fused patch might expand and gain membrane area and thereby relax the membrane tension or the adhering hemifused state might remain stable. *Grafmüller et al*. found that the fusion process consists of at least three consecutive steps in which the first two steps are tension dependent: (1) Interbilayer flips of lipid tails, (2) nucleation of a small hemi-fused area and (3) pore formation. Interbilayer flip and nucleation of hemi-fusion depend both exponentially on the tension. It is conceivable that the hydrophobic contact is favoured at intermediate tension facilitating interbilayer exchange (lipid splay) of lipids in the opposing membranes. Using simulations that enforce interbilayer flips of individual lipid tails and utilizing Jarzynski’s relation, *Grafmüller et al*. determined the energy scale for these barriers^37^.

According to *Kozlov* and *Chernomordik* three major origins of forces acting on cell membranes and generating membrane tension exist: (1) osmotic pressure, i.e., a difference in hydrostatic pressure between the cytosol and the external medium; (2) membrane adhesion to the actomyosin cortex and (3) cellular adhesion and subsequent spreading on substrates or other cells^18^. The characteristic tensions found in the plasma membrane of eukaryotic cells are only in the range of 0.01–1 mN/m depending on the cell type. Therefore, typical tension values found in the plasma membrane of cells are maybe not sufficient to substantially increase the fusion efficiency. This is rather expected, considering the fact that unwanted fusion needs to be suppressed in non-specialized cells. Proteins from the active zone could in principle enhance fusion probability by increasing tension locally very close to rupture tension. *Martens* *et al*. found that, synaptotagmin-1 promotes SNARE-mediated fusion by lowering the associated activation barrier by inducing high positive curvature in target membranes by C2-domain membrane insertion. Thereby, synaptotagmin-1 is believed to trigger the fusion of docked vesicles by local buckling of the plasma membrane increasing the tension in the outer monolayer and thus the hydrophobicity, which reduces relevant energy barriers^38^.

Such increased tension would need to be locally constrained since tension inhibits clathrin mediated endocytosis of synaptic vesicles. Recently *Wen et al*. found that dynamic assembly of filamentous actin including also ATP hydrolysis, mediates Ω-profile merging by providing sufficient plasma membrane tension to shrink the Ω-profile in neuroendocrine chromaffin cells containing ~300 nm vesicles. It is clear that mechanical tension plays a pivotal role in vesicle trafficking and it will be interesting to see in the future how cells regulate tension to adjust the energy landscape of these processes in order to control their kinetics.

## Conclusion

Theoretical predictions and computer simulations suggested that intermediate membrane tension might reduce energy barriers of membrane merging and increase the probability of fusion. However, no direct experimental evidence for these claims existed. Here, we devised two methods to laterally dilate membranes in a defined way. Firstly, we used isolated membrane patches derived from GUVs spread on a stretchable PDMS sheet to exert a constant tension in the membrane. Secondly, sessile GUVs were set under tension by the adjusting adhesion energy. We found that at intermediate tension, SNARE-mediated fusion efficiency is indeed increased substantially, which we attribute to facilitation of lipid splaying and assisted fusion pore expansion. The results prove experimentally the theoretically predicted hypothesis that membrane tension steers membrane fusion and might therefore be an essential prerequisite for fast fusion as observed for the release of neurotransmitters at the neuronal synapse. The implications of our study are that local tension needs to be substantial, even close to bilayer lysis to increase fusion efficiency with unstressed vesicles. However, if synaptical vesicles additionally being exposed to curvature stress meet pre-stressed planar membranes fusion rates observed in nature might be reached also with model membranes.

## Materials and Methods

### Design of membrane stretcher

The membrane stretching device was designed with CAD software (Solid Edge: Siemens PLM Software) and consists of a stretchable PDMS (poly-dimethyl siloxane, Sylgard 184, Dow Corning) membrane on top of a milli-structured layer (Fig. 1). The PDMS layer has one central channel (3 mm wide, 1 mm high, 20 mm long) and two side channels (1 mm W, 1 mm H, 16 mm L). By applying negative pressure within the side channels, the elastic walls deflect and thereby stretch the membrane in the central channel. The thickness of the layer and the PDMS sheet is about 5 mm and 180 μm, respectively. See Supplementary Figure 1 for a photograph of the device.

### Fabrication of membrane stretcher

The membrane stretcher was produced by replica moulding^39^. The PMMA (poly-methyl methacrylate) mould master was fabricated with a CNC milling machine (DMC 1035, DECKEL MAHO). The PDMS prepolymer was mixed with curing reagent (10:1 w/r), poured over the mould master, placed in a vacuum chamber for degassing and baked at 75 °C for 45 min. The solidified PDMS layer was peeled off from the master and bound subsequently to the PDMS membrane.

### Lipid composition

The lipid membrane of the giant unilamellar vesicles (GUVs) and large unilamellar vesicles were composed of the following phospholipids: 1,2-dioleoyl-*sn*-glycero-3-phosphocholine (DOPC), 1,2-dioleoyl-*sn*-glycero-3-phosphoethanolamine (DOPE), 1,2-dioleoyl-*sn*-glycero-3-phospho-l-serine (sodium salt) (DOPS) (Avanti Polar Lipids, Alabaster, AL, USA), and 3β-hydroxy-5-cholestene (Cholesterol) (Sigma-Aldrich, St. Louis, MO, USA). The lipid fluorophores Atto390-DOPE (A390), Atto488-DOPE (A488) and Atto594-DOPE (A594) were purchased from ATTO-TEC GmbH (Siegen, Germany). Lipid films of different compositions were obtained by mixing stock solutions (1–10 mg mL^−1^) in chloroform and evaporation of the solvent under nitrogen flow. To remove residues of the organic solvent the lipid films were dried in a vacuum oven at 30 °C for 2 h. GUVs were made from a lipid film with a composition of DOPC/DOPE/DOPS/Cholesterol/lipid fluorophore (55:22:11:11:1) mol% to mimic the natural inner leaflet composition of lipid headgroups in mammalian cells. The composition was also used in other vesicle fusion assays^40^. For the GUV-adhesion measurements, 2 mol% of 1,2-dioleoyl-*sn*-glycero-3-phosphoethanolamine-N-(cap biotinyl) (cap-biotinyl-DOPE) replaced 2 mol% of DOPE in the lipid composition to introduce a specific binding interaction of GUV-membranes to avidin coated glass surfaces. LUVs were prepared with the same composition except that the lipid dye was either ATTO 594-DOPE or ATTO 488-DOPE.

### Buffers

HEPES-buffer A (20 mM, 2-[4-(2-hydroxyethyl)piperazin-1-yl]ethanesulfonic acid, equipped with 98.5 mM KCl, 1 mM ethylenediaminetetraacetic acid, 0.1 mM dithiothreitol, pH 7.4) was used in the vesicle fusion assay on SLBs. HEPES-buffer B (15 mM, HEPES, equipped with 67.5 mM KCl, 0.1 mM dithiothreitol, pH 7.4) was used for the GUV-adhesion experiments. The concentration of magnesium chloride (up to 10 mM) in HEPES-buffer B was increased in some GUV-adhesion experiments and the HEPES concentration reduced at once (67.5–52.5 mM) to maintain the osmolality of (150 ± 2) mosmol kg^−1^. Moreover, to facilitate the spreading of GUVs that adhere to the hydrophilic PDMS surface, a high ionic strength buffer (20 mM HEPES, 300 mM NaCl, 0.1 mM EDTA, pH 7.4) was used to create a hyperosmotic environment. A phosphate-buffer (137 mM NaCl, 3 mM KCl, 10 mM Na_2_HPO_4_, 2 mM KH_2_PO_4_, pH 7.4) with bovine serum albumin (0.1 mM) was used for the passivation of the hydrophilic PDMS surface. All buffers were made from pure water of a MilliQ system (EMD-Millipore, Merck Darmstadt, Germany) filtered through a cellulose-acetate-membrane (Minisart, Sartorius, Göttingen, Germany) and eventually degassed. The osmolality of each buffer was controlled with an osmometer (Osmomat 3000, Gonotec, Berlin, Germany).

### Protein purification

Proteins were overexpressed in *E*. *coli* BL21 (DE3) as N-terminal 6 × -His tagged versions using pET vectors (Novagen) and affinity-purified using Ni^2+^-nitrilotriacetic acid (NTA) agarose (Qiagen) resin followed by thrombin cleavage to remove the 6 × -His tags. The proteins were further purified by ion-exchange chromatography using the ÄKTA system (GE Healthcare). The ΔN-complex (ΔN) was formed by mixing Syntaxin1A (183–288), SNAP-25 (1–206) and Syb2 (49–96) in the molar ratio = 1:1:1.5 and purified as described earlier^31,41^. Full-length synaptobrevin (1–116) (Syb) was also purified as described earlier^42^. Purified proteins were snap-frozen with liquid nitrogen and stored at − 80 °C until use.

### Reconstitution of SNAREs

The reconstitution of the synaptobrevin or ΔN-complex into lipid vesicles was performed as previously described by *Schwenen et al* ^34^. Accordingly, lipid films (630 nmol) were dissolved in HEPES-buffer (50 µL) containing n-octyl-β-D-glucopyranoside (NOG) (100 mM) and incubated for 30 minutes to yield a solution of micelles. Both protein stock solutions contained 1% CHAPS. The micelle solution and proteins were mixed and incubated for 30 minutes on ice. GUVs were prepared with the ΔN-complex resulting in a protein to lipid ration of around 1/350, LUVs with synaptobrevin (2 nM) resulting in a protein to lipid ration of around 1/1400. To remove the detergent molecules a Sephadex column (illustra NAP-25, GE Healthcare, Chicago, USA) was prepared with HEPES-buffer. After elution of the micelle-protein mixture, the large unilamellar vesicles (LUV) (50–900 nm in diameter) containing SNARE-proteins were collected in a reaction tube. Concentrating the vesicle solution in a vacuum centrifuge (Concentrator 5301, Eppendorf, Hamburg) to a volume of 80 to 150 µL and elution in a column with pure water result in an ion free vesicle solution that was concentrated to a final volume of around 100 µL. The solution with synaptobrevin-containing vesicles was poured into a small reaction tube and dried in a desiccator filled with a saturated sodium chloride solution. LUVs were obtained by resuspending the lipid film in the reaction tube for 30 minutes with HEPES-buffer. Small droplets (2 µL) containing LUVs with the ΔN-complex were administrated onto ITO-slides that also were dried in the desiccator. Between the two ITO-slides a PDMS spacer was placed, and small pieces of copper were glued at the opposite edge of each ITO-slide. The inside of the chamber was filled with sucrose solution (150 mOsmol/kg) and a sinusoidal voltage of 1.6 Vpp with a frequency of 12 Hz was applied for 2.5–3 h to obtain GUVs with a size of about 3–50 µm in diameter. The GUV- and LUV-solution can only be stored on ice for a maximum of one day. The size distribution of the LUVs was 50 nm ‒1 µm as determined by dynamic light scattering (Zetasizer Nano S, Malvern Instruments, Malvern, UK). For details, see Supplementary Figure S27.

### Confocal laser scanning microscopy

Confocal microscopy experiments was performed with a BX61 upright and IX83 inverted microscope combined with a FV1200 CLSM unit (Olympus, Tokio, Japan) equipped either with a 60 × water immersion objective (Olympus) or a 60 × oil immersion objective (Olympus). LASERs with wavelengths of 405 nm, 488 nm and 561 nm were used in a non-sequential mode with a scanning rate of 2 µs per pixel. Images with a size of 1600 × 1600 pixels were recorded, with pixel size of 0.132 × 0.132 µm^2^ for the experiments with the SLBs on a PDMS surface. Image stacks of adhered GUVs on a functionalized glass surface were recorded with different pixel sizes near the diffraction limit.

### Preparation of membrane coated PDMS surface

The surface of the thin PDMS layer of the chamber was oxidized with an oxygen plasma for ten seconds using a Zepto plasma generator (Diener, Ebhausen, Germany)^43^. Only the inner part of the PDMS network that can be stretched was oxidized. Aluminium foil was placed around that area to protect the other surfaces from being oxidized. Directly after the plasma oxidation, 500 µL of HEPES-buffer were added onto the surface to maintain the hydrophilic character of the PDMS. Spreading of GUVs on the hydrophilic PDMS surface was accomplished by incubating the GUV-solution for a few minutes to form islands of independent (non-connected) and defined membrane areas. Passivation of the uncovered hydrophilic PDMS surface was achieved by incubation of the PDMS surface with albumin solution in phosphate buffer for 15 minutes (Fig. 1B).

### LUV fusion on PDMS-based lipid bilayers

The side channels of the PDMS substrate were connected via a flexible tube and luer lock connectors to a gas tight syringe (10 mL, Hamilton, Reno Nevada, USA) that was controlled by a syringe pump (Fusion 200, KR Analytical, Sandbach Cheshire, UK) (Fig. 1). After preparation of supported lipid bilayers (SLBs) on the PDMS, the syringe pump generated a reduced pressure in the connected tube and PDMS channel system by withdrawing the stamp of the syringe (10 mL, Hamilton, Reno, NV, USA) for 1–4 mL. A lower pressure induced a small stretch of the PDMS surface between the side channels (Fig. 1). With this system a dilation between 0–4% can be achieved, which was determined through the change in distance between quantum dots that were incorporated into the thin PDMS (Supplementary Information Figure S2). For the analysis of the membrane area the minimum cross entropy thresholding technique by *Li and Tam* implemented in the open source software ImageJ was used^19^.

LUVs were incubated for 30–45 minutes on stretched PDMS surfaces displaying membrane patches from ruptured GUVs. Fluorescence images of relaxed and stretched membrane patches were taken, for comparison. LUV fusion was detected by the increase of fluorescence emitted from the lipid dye A594 newly occurring in the membrane patches on the PDMS substrate after lipid mixing. We employed an intensity threshold as shown exemplarily in Fig. 2B to avoid counting adsorbed vesicles.

LUVs were classified as docked if they do not lose fluorescence intensity, do not shrink and at the same time do not display fluorescence emitted from the blue dye A390. Hemifused vesicles also do not shrink but show additional fluorescence emitted from A390 that was originally only present in the planar membrane patch.

### Preparation of adhered giant unilamellar vesicles

A hydrophilic glass surface (MatTek Corporation, Ashland, MA, USA) was functionalized with avidin (1 µM, in PBS) to create binding sites on the surface for biotinylated lipids. After coating of a hydrophilic glass surface with phosphate-avidin buffer solution, a phosphate buffer equipped with casein (100 µM) or bovine serum albumin (100 µM) was used to passivate the potentially uncovered glass surface. For the adjustment of the adhesion area of the GUVs on the surface, Mg^2+^-ions in a concentration range of 0.2–6 mM were added. The used HEPES-buffer was adjusted to an osmolality of (150 ± 2) mOsmol kg^−1^ to prevent osmotic stress acting on the GUV-membrane. Evaporation of water from the buffer solution from the sample petri dish was prevented by sealing it under a dome at the used microscope during the measurements. A small volume (20–100 µL) of the electroformed GUVs was added to the prepared sample solution. The GUVs settled onto the surface and adhered with a defined adhesion area that increases with higher Mg^2+^-ion concentrations. Over a time period of around one hour, the adhered GUVs were stable as far as shape and adhesion zone were concerned.

### LUV fusion to adhered giant unilamellar vesicles

LUVs were added to the pre-stressed GUVs using a syringe. Between 3–50 minutes after the addition of LUVs a three-dimensional image stack of the adhered GUVs was created to quantify the number of docked LUVs and determine the fusion efficiency. The fusion efficiency for the GUVs was computed according to Eq. (3) substituting *I*_patch_ against *I*_adhesion_, which is the fluorescence intensity of the LUV dye at the contact site of the GUV with the substrate. The fluorescently labelled dye A488 was used to stain the LUV membrane, whereas A594 was present in the GUV membrane. In some cases, A594 was used to label the LUV membrane and A390 to label the GUV membrane to avoid FRET effects.

### FRAP measurements

Fluorescence recovery after photobleaching (FRAP) was carried out to confirm LUV fusion on SLBs and GUVs. Therefore, the lipid dye from the LUVs was bleached with full laser intensity for a few seconds and images were taken to record the recovery of the dye into the bleached spot. FRAP-measurements on SLBs and GUVs are shown in the supplementary information (Figures S9–S12, S21 and S22).

### Area scans

The area scan shown in Fig. 2 was normalized to the mean intensity of the dye A390 present in the SLB. Both channels for the dyes A390 and A594 were normalized to the mean grey value of A390 in the SLB patches as a reference.

## Electronic supplementary material

Supplementary information
