# Supplementary material for: Membrane tension increases fusion efficiency of model membranes in the presence of SNAREs
Source: Sci Rep. 2017 Sep 21;7:12070. doi: 10.1038/s41598-017-12348-w (PMC5608890; doi:10.1038/s41598-017-12348-w)
Supplement: Supplementary file 1 — Supplementary information [file 41598_2017_12348_MOESM1_ESM.pdf]

Supplementary Information

Membrane tension increases fusion efficiency of model membranes in the presence of SNAREs

Torben-Tobias Kliesch, Jörn Dietz, Laura Turco, Partho Halder, Elena Alexandra Polo, Marco Tarantola, Reinhard Jahn, Andreas Janshoff*

Institute of Physical Chemistry, Georg-August-University, Göttingen 37077, Germany

Max-Planck-Institute for Dynamics and Self-Organisation, Göttingen 37077, Germany

Department of Neurobiology, Max-Planck-Institute for Biophysical Chemistry, Göttingen 37077, Germany

Section 1: Production of the PDMS device

The base and curing agent mixture (3.0 g/0.3 g) was degassed under vacuum for 30 minutes and cast onto the silicon wafer that was placed on a spin coating system (SCS G 3 Spin Coater Series, Special Coating Systems, Indianapolis, IN, USA). For the production of a very thin PDMS (polydimethylsiloxane) layer, the spin coating system rotated the silicon wafer with 500 rpm for 30 seconds with a starting and ending ramp of ten seconds. Then the wafer with the fluid was placed in an oven preheated to a temperature of 75 °C for 45 minutes to obtain a very thin, polymerized PDMS layer. The thickness of these layers was 180 µm measured with a bright field microscope BX51 (Olympus, Tokio, Japan) equipped with a CCD-camera (DP71, Olympus). Subsequently, the previously prepared milli-structured layer was placed on a wafer with a fluid mixture of the base and curing agent for a few seconds. Then it was placed on the wafer with the cured, thin PDMS layer. Again, the wafer was heated to 70 °C for 30 minutes in an oven. This procedure leads to a solid connection between the thin PDMS layer and the PDMS-chamber. With a scalpel, the thin PDMS sheet was cut out and the whole part with the thin PDMS sheet on top could be removed carefully by hand yielding a smooth surface on top of the thin PDMS sheet. Due to its soft and flexible nature, the thin PDMS sheet could be stretched up to a few percent of its initial area until it ruptures and cracks occur. Figure S1 shows the used PDMS stretching device.

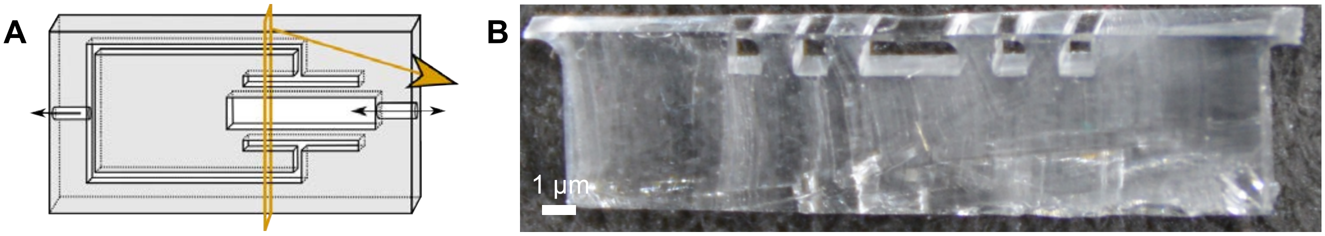

Figure S1 | Cross-sectional view of the substrate. A) Scheme of the PDMS stretching device. B) A cross-sectional view shows the channel system and spanned PDMS layer on top. In the centre, a big channel is visible that is connected to the surrounding air. The free standing PDMS sheet is around 180 µm thick. The channels to the left and to the right are referred to as the side chambers. Here, vacuum can be applied to stretch the PDMS sheet. The outer channels are the connection to the syringe pump system (see A)).

Section 2: Dilatation of supported lipid bilayers on hydrophilic PDMS surface

Supported lipid bilayers (SLBs) on hydrophilic surfaces are produced by spreading of vesicles. SUVs and LUVs normally form bilayers that fully cover the surface of the substrate if the appropriate vesicle concentration in the solution is used.

As depicted in Figure S1, the membrane-covered PDMS sheet was stretched to reach a defined area dilatation of the surface. Fluorescent beads incorporated into the thin PDMS sheet were used to document the local area dilatation. Figure S2 shows the dilatation of a membrane fully covering the PDMS surface. Green fluorescent beads were incorporated into the thin PDMS sheet. Applying lower pressure to the adjacent side channels of the PDMS layer leads to dilatation of the SLB firmly attached to the PDMS sheet. Prior to stretching, the surface is completely covered with a membrane (red fluorescence) without displaying any wrinkles or cracks. Figure S2 B shows the surface after applying vacuum to the side channels. The stretching of the PDMS surface results in a surface dilatation of 4.1%, which is slightly above the maximal area increase for this lipid composition of 3.6%. Therefore, it was possible to observe small cracks in the membrane. The image in figure S2 C shows a ruptured membrane on the stretched PDMS-support. Vertical cracks are aligned in parallel. At this stage, the surface dilatation was around 7.6%, which is even beyond the predicted area-increase at lysis tension. Releasing the surface dilatation by increasing the air pressure in the side channels, yields back a relaxed membrane. In the left part of the picture in figure S2 D small membrane tubes emerge indicating that the SLB area is now compressed. Tube formation can be explained by a slightly compressed surface. The membrane tube formation also confirms membrane fluidity because the lipids in the membrane are mobile and can form tubes through compression.

This documentation of dilating SLBs on the fabricated PDMS surface of the membrane stretcher device shows that it is possible to dilate membranes between 0 - 7.6% of their initial area, which can be measured by fluorescence microscopy. Cracks and defects in the SLB occurred at around 4% area increase and could be clearly identified in the fluorescent images.

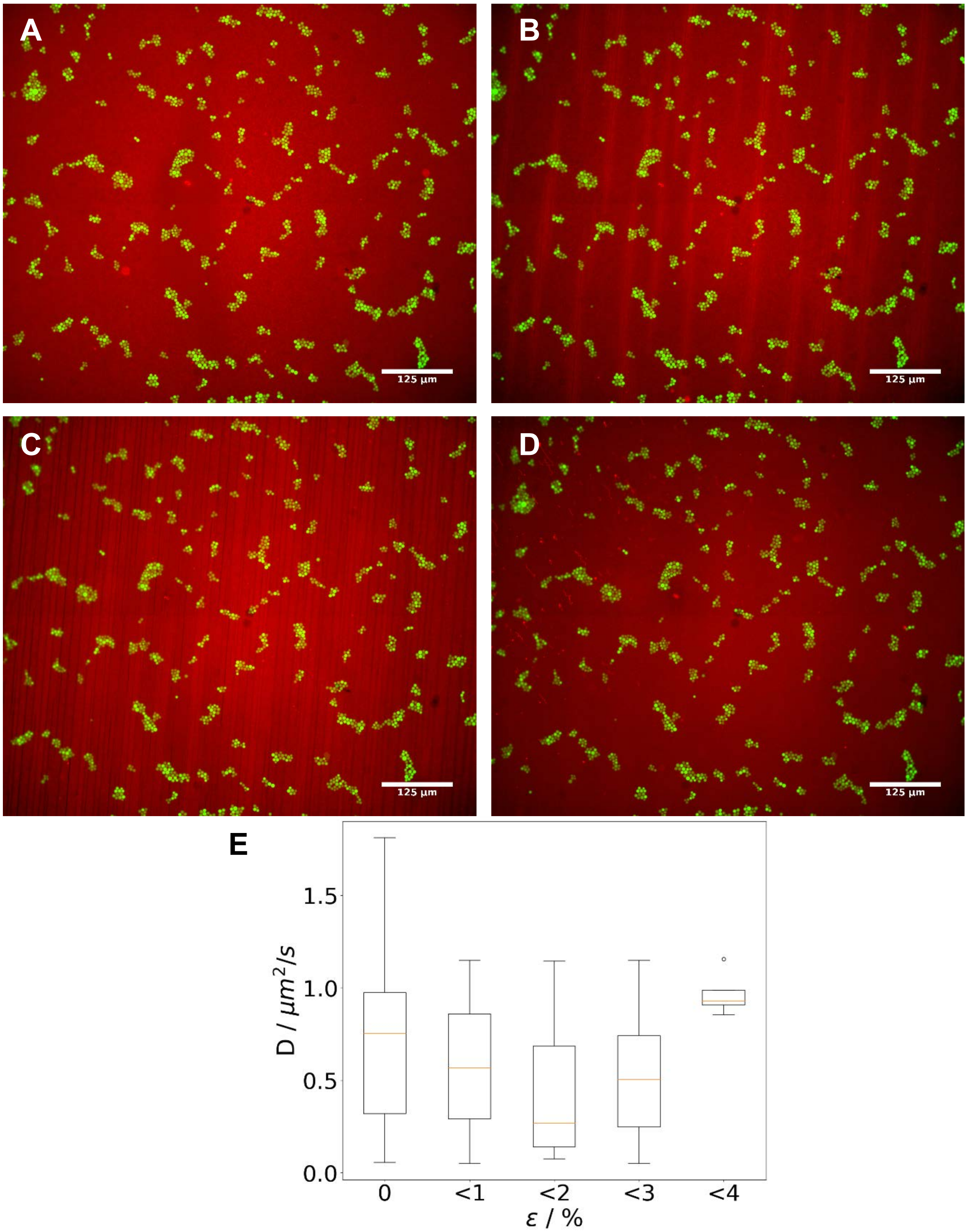

Figure S2 | Dilatation of PDMS surface fully covered with a lipid bilayer. A) The SLB (red) covers the entire PDMS surface. Green fluorescent beads were incorporated into the thin PDMS sheet below the surface to measure the dilatation. B) The uniaxial dilatation between the beads at the left and right was around 4% and the SLB on the surface started to rupture. C) Vertical cracks in the SLB occur because of the high area dilatation of around 7.6%. D) Relaxing of the PDMS surface led to compression of the SLB and formation of membrane tubes visible at the left side of the image. E) Diffusion constants of a lipid bilayer deposited on stretchable PDMS as a function of strain ε.

Passivation of PDMS surface

LUV fusion to SLBs and spreading of the LUVs on the PDMS substrate was tested with a sample without any further passivation of the substrate surface around the membrane patches and in the absence of SNARE proteins. Figure S3 shows two images representing the two fluorescently labeled lipids, A594 in the SLBs (A) and A488 (green) in the LUVs (B). The right image reveals that LUVs are everywhere but on the membrane patches documenting that it is necessary to passivate the surface to prevent unwanted LUV adsorption and subsequent fusion at the edges of the SLBs as well as spreading of the LUVs onto the hydrophilic PDMS surface. As a consequence, the protein BSA was used to protect the hydrophilic PDMS surface.

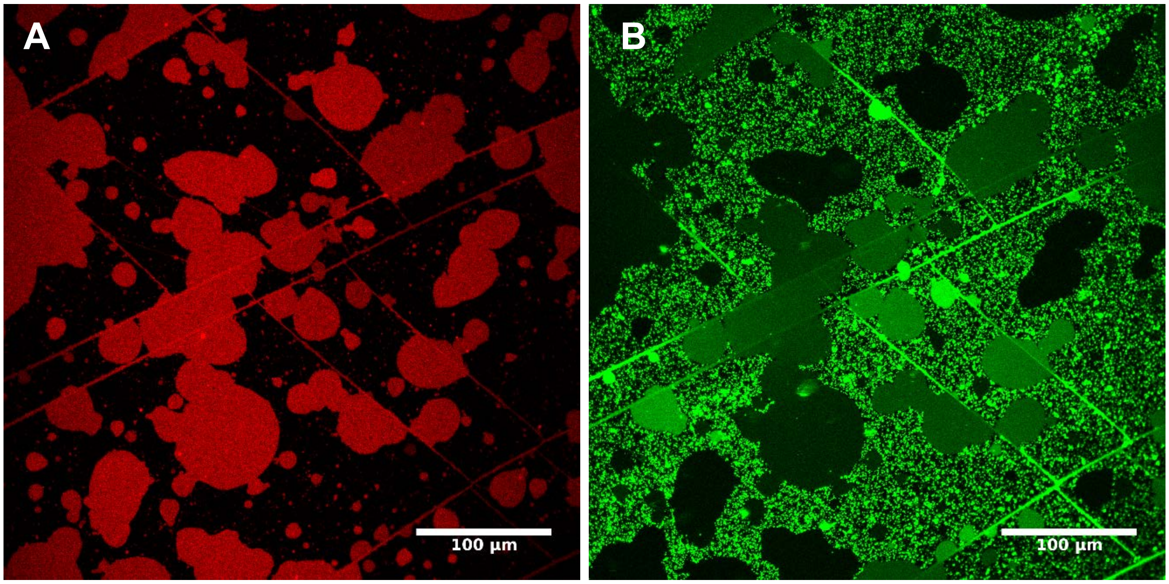

Figure S3 | Control sample without passivation. A) Spread GUVs on the PDMS surface that from SLBs (red). B) Addition of LUVs (green) in the absence of SNAREs. LUV adsorb on the uncovered surface and even might fuse with SLBs. Cracks in the PDMS surface also promote LUV fusion to the SLBs.

Control experiments with passivated surfaces in the absence of SNAREs

Figure S4 shows the three different control experiments ruling out non-specific fusion. In figure S4 A an experiment is shown where the membrane patches on the stretched PDMS surface lack SNARE proteins but the added LUVs are equipped with the ΔN49-complex, composed of syntaxin and SNAP25. Even after stretching of the PDMS surface (2 mL vacuum) no membrane fusion or docking of the LUVs to the membrane patches could be observed after more than 30 minutes of incubation.

In figure S4 B, a control sample without any area change of the membrane patches is shown. The membranes were equipped with SNARE proteins on both membrane sites, GUV (ΔN49) and LUV (Syb). This control sample shows no increase in fluorescence intensity originating from LUV fusion after 30 minutes of incubation time documenting that fusion requires tension. In rare cases, larger LUVs fuse with the membrane patch.

In figure S4 C two membrane patches (blue) are shown after incubation of LUVs (red) in the absence of SNARE proteins in the sample. Additionally, the PDMS surface was dilated (2 mL vacuum) but no LUV fusion on membrane patches could be detected. The corresponding fluorescence intensity area or line scans for the SLB dye A390 and LUV dye A594 are shown on the right-hand side.

The data shows that passivation of the hydrophilic PDMS surface around the membrane patches with BSA was successful to prevent undefined fusion events and that SNAREs in combination with pre-stressed membranes are necessary to trigger fusion.

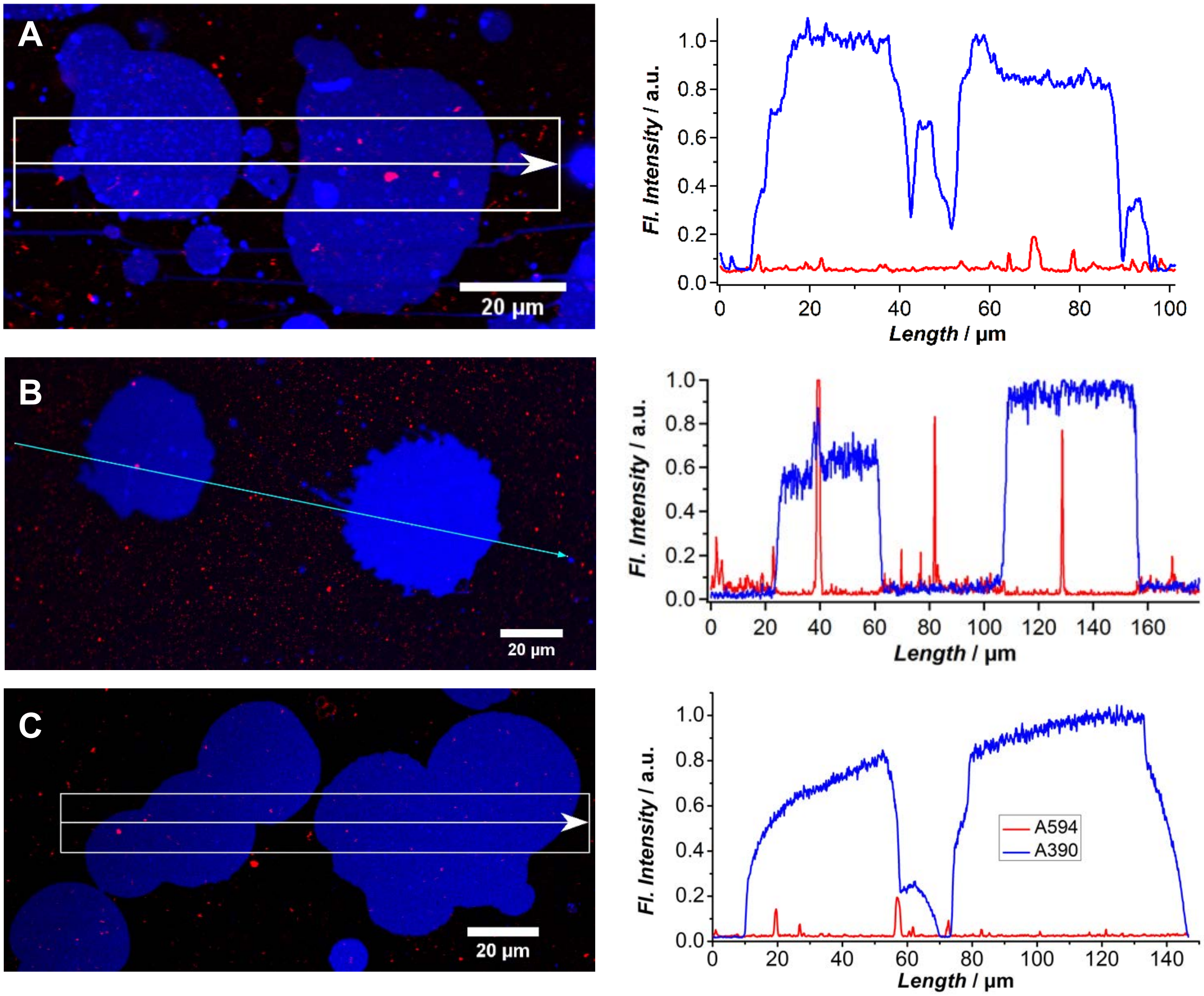

Figure S4 | Control sample without SNARE proteins on one or both membrane sites. A) Stretched membrane patches without SNAREs are shown together with LUVs containing the ΔN-49 complex (Syntaxin preassembled with SNAP25). The area scan on the right displays the average fluorescence intensity of both dyes confirming that LUVs do not fuse or dock to the tensed SLBs. B) Control sample with SNAREs but without significant membrane area dilatation shows no fluorescence intensity increase of the LUV dye at the SLBs after an incubation time of 30 minutes. C) Fusion experiment using a pre-stressed membrane and LUVs in the absence of SNAREs. The two-channel image (blue SLBs: A390; red LUVs: A594) shows only a few LUVs docked to the SLBs and on the passivated PDMS-surface. LUV fusion on the SLBs was absent because there was no detectable fluorescence increase of the LUV dye A594 on the SLBs.

Section 3: SNARE-mediated LUV fusion experiments on stretched SLBs

Figures S5-S8 correspond to the experiments 1-4 compiling all 30 membrane patches used for the analysis of tension-dependent fusion. The area-change of each SLB due to the stretching of the substrate was measured with the thresholding technique of *Li et al.* being part of the image processing software ImageJ. The left picture shows the membrane patches in the channel for the dye A390 after stretching and subsequent LUV incubation. The picture on the right shows the red channel corresponding to emission of the LUV dye A594. The LUV dye intensity varies on each membrane patch and depends on the SLB area increase after stretching of the PDMS. Figure S5 shows 13 membrane patches with only moderate LUV adhesion on the PDMS surface compared to the experiments 2-4 shown in figure S6-S8. A mean relative LUV dye intensity increase of A594 was measured on each membrane patch by thresholding of the channel for A390 and selecting the corresponding red ROI for the membrane patch. This ROI was used in the red channel (A594) to provide the mean intensity $I_{LUV,\ A594}$ on the membrane patch. The fluorescence intensity I_{max} of the dye A594 on an adhered LUV on the PDMS surface was taken as a reference. The fusion efficiency F_{eff} was calculated from the channel of the dye A594 for every membrane in all images pictures that were taken after the LUV incubation.

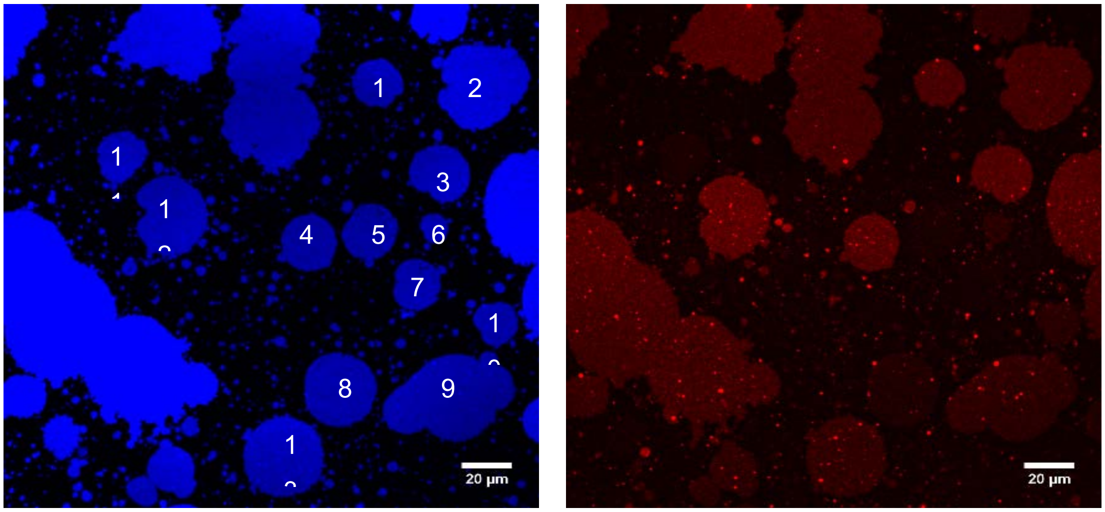

Figure S5 | Experiment 1: Image of membrane patches after stretching (left, A390) and incubation for 40 minutes with LUVs (right, A594).

In figure S5 the membrane patches of experiment 1 are displayed after PDMS dilatation and subsequent LUV incubation. SNAREs were used as membrane fusion promoters as detailed in the main text. Some membrane patches have more LUV dye content compared to others. The mean fluorescence intensities of the LUV dye A594 differs between the membrane patches. In the centre, five membrane patches (3-7) were selected to illustrate and explain the fusion efficiency of LUVs on membrane patches with and without an area increase compared to their initial area.

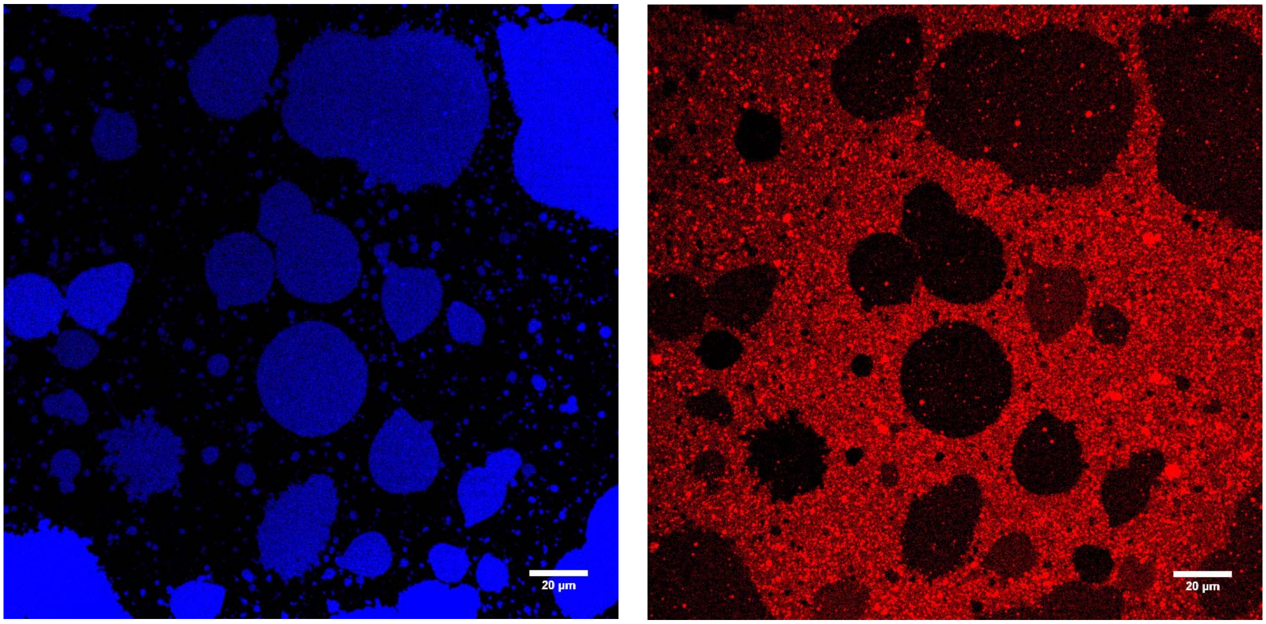

Figure S6 | Experiment 2: Images of membrane patches (left, A390) after LUV-incubation (right, A594).

In figure S6 (left image) the membrane patches at the border emit more fluorescence since these patches were not within the previously scanned area and thus were not subject to photobleaching as much as those in the central region. The image on the right-hand side shows the fluorescence intensity of the LUV dye A594. Many LUVs adhere non-specifically to the PDMS surface. Also in this image, some membrane patches appear brighter in the red channel of dye A594 indicative of fusion with the membrane patches.

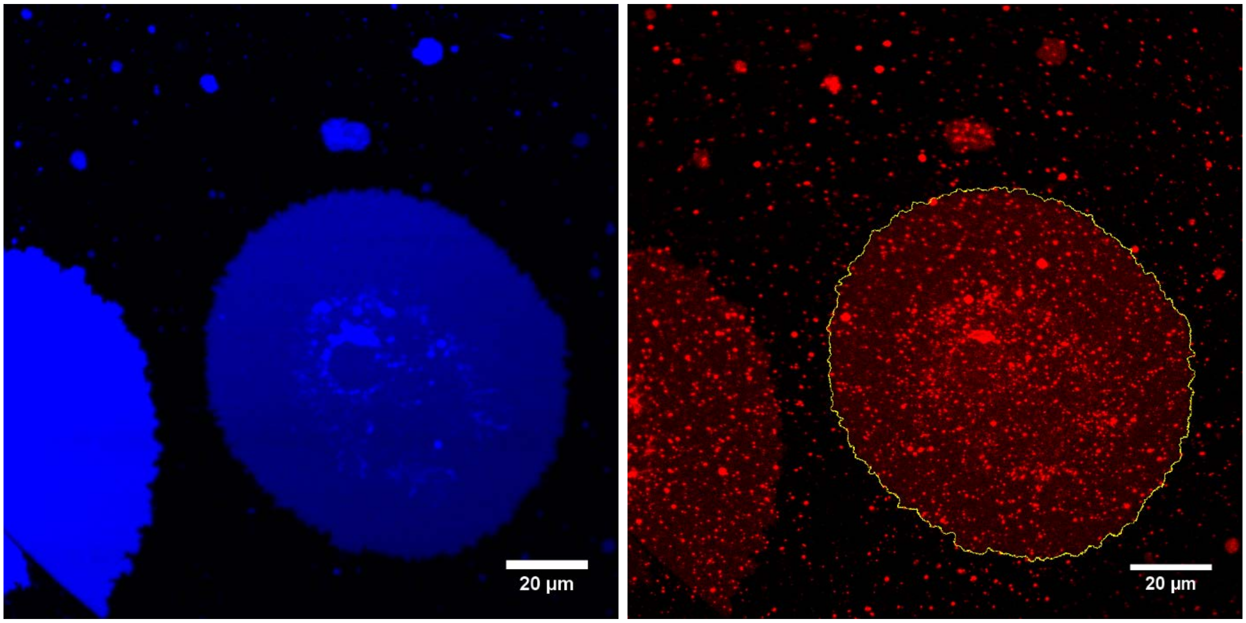

Figure S7 | Experiment 3: A very large membrane patch showing an area change of (1.4±0.1)% after stretching the underlying PDMS substrate provoked a large amount of docked and fused LUVs (red dye A594). Docked vesicles appear as bright red spots, while fused LUVs generate a homogeneous fluorescence in the membrane patch.

Figure S7 shows a single large membrane patch with a size of 6246 μm^2 after stretching of the PDMS sheet and after addition of LUVs. For this membrane patch an area change of (1.4±0.1)% was found leading to a fusion efficiency of (5.8±0.1)%.

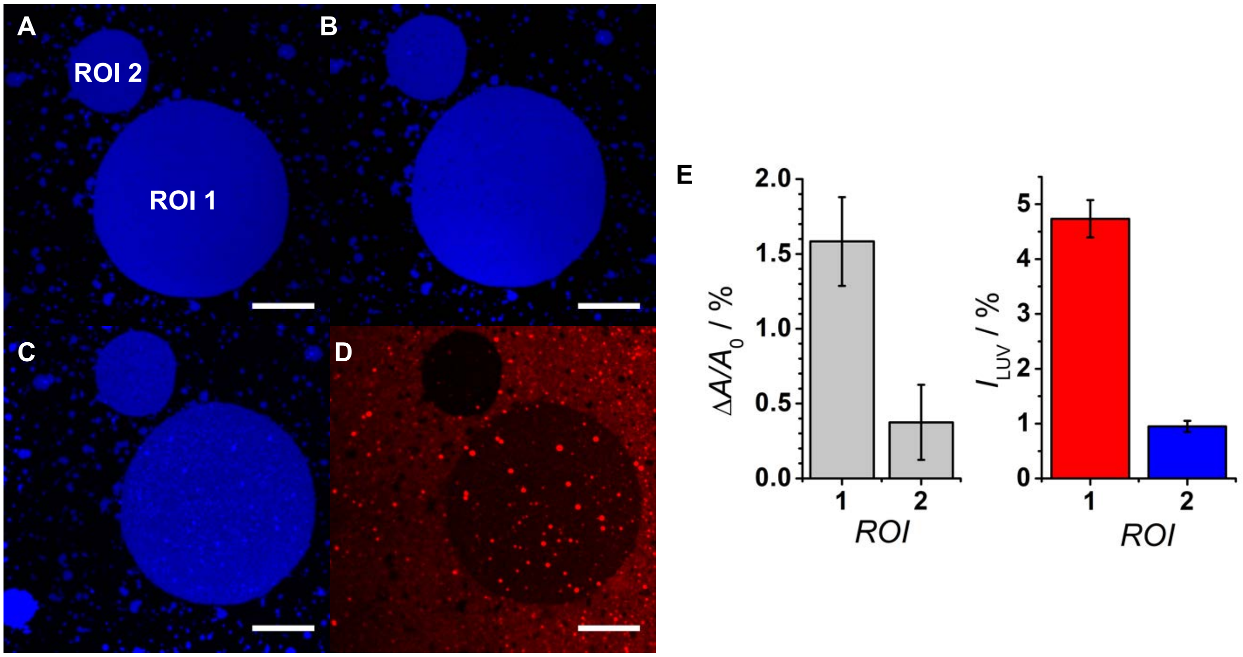

Figure S8 | Experiment 4: A) Two unstressed membrane patches. B) Stretching of the PDMS substrate led to a strong increase in area of ROI 1 but only a small area-change of ROI 2 was observed. Upon stretching small holes appear in the supported bilayers. C) Addition of LUVs (A594) to the pre-stressed membrane patches. Fusion of LUVs with the membrane patches was predominately found with ROI 1. D) Fluorescence image of the red channel (A594) after incubation with LUVs. The LUVs showed a higher affinity to the stretched ROI 1. E) The measured membrane area changes and relative fluorescence intensities $I_{LUV} = F_{eff}$ are shown. Scale bar: 20 μm.

In figure S8 two membrane patches were shown that exhibit a different membrane tension after PDMS dilatation. Holes in the membrane were subtracted prior to area determination. The membrane patch labelled as ROI 1 exhibits an area increase of (1.6±0.3)% after stretching of the PDMS substrate and shows a much higher fusion efficiency F_{eff} = (4.7±0.3)% compared to the membrane patch labelled ROI 2 ($\Delta A/A_0$ = (0.4±0.3)%, F_{eff} = (1.0±0.1)%). Docking, hemi-fusion and fusion of LUVs occurs more frequently on ROI 1.

FRAP measurements of PDMS-based SLBs

Experimentally, a high-energy LASER pulse was used to bleach a region of interest (ROI) on the supported lipid bilayer with the actual bleaching area radius r. The diffusion coefficient D can be obtained from equation (I),[1] where r is the bleaching radius of the LASER and $t_{1/2}$ the half-time recovery for the intensity:

$$D = 0.224 \cdot \frac{r^2}{t_{1/2}}. \qquad \text{(I)}$$

In figures S9-S10 FRAP measurements performed on a SLBs were shown. It was ensured that indeed LUV fusion occurred by bleaching the lipid dye A594 to monitor the mobility of the newly inserted dye. A fast recovery occurred on the SLBs with only a small immobile fraction (15%) and a diffusion coefficient of (0.9±0.1) $\mu m^2\ s^{-1}$.

Figure S10 shows the fluorescence recovery of bleached LUVs that are still attached to the SLB. The FRAP measurement confirms a shared mono- or even bilayer. The largest and therefore brightest vesicle in figure S10 A visible in the in the yellow circle only showed a very minor recovery after bleaching, which might be indicative of mere docking (Fig. S10 C).

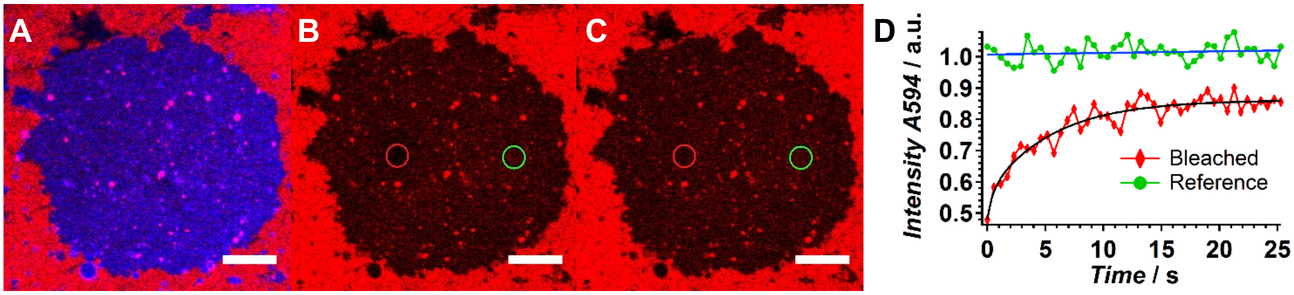

Figure S9 | FRAP measurement performed on a membrane patch (SLB) after fusion with LUVs. A) Overlay of red and blue channel showing the membrane patch (blue) after the incubation with LUVs. B) Bleaching of the red dye A594 within the area of the red circle with full LASER power. The enclosed area indicated by the green circle was used as a reference. The background fluorescence stems from non-specifically adsorbed LUVs. C) After approximately 20 seconds, the diffusion of the LUV dye A594 back into the bleached spot was complete. D) Intensity recovery as a function of time shows an immobile fraction of 15%. Scale bar: 10 µm.

[1] Soumpasis, D. M., Theoretical analysis of fluorescence photobleaching recovery experiments. *Biophys. J.* **1983,** *41* (1), 95-97.

Bleaching of the edges of the SLBs and the LUVs that adhered to the PDMS surface (Figure S11) proved that LUVs adhere to the PDMS but do not spread to form a lipid bilayer because no recovery of the bleached area could be detected.

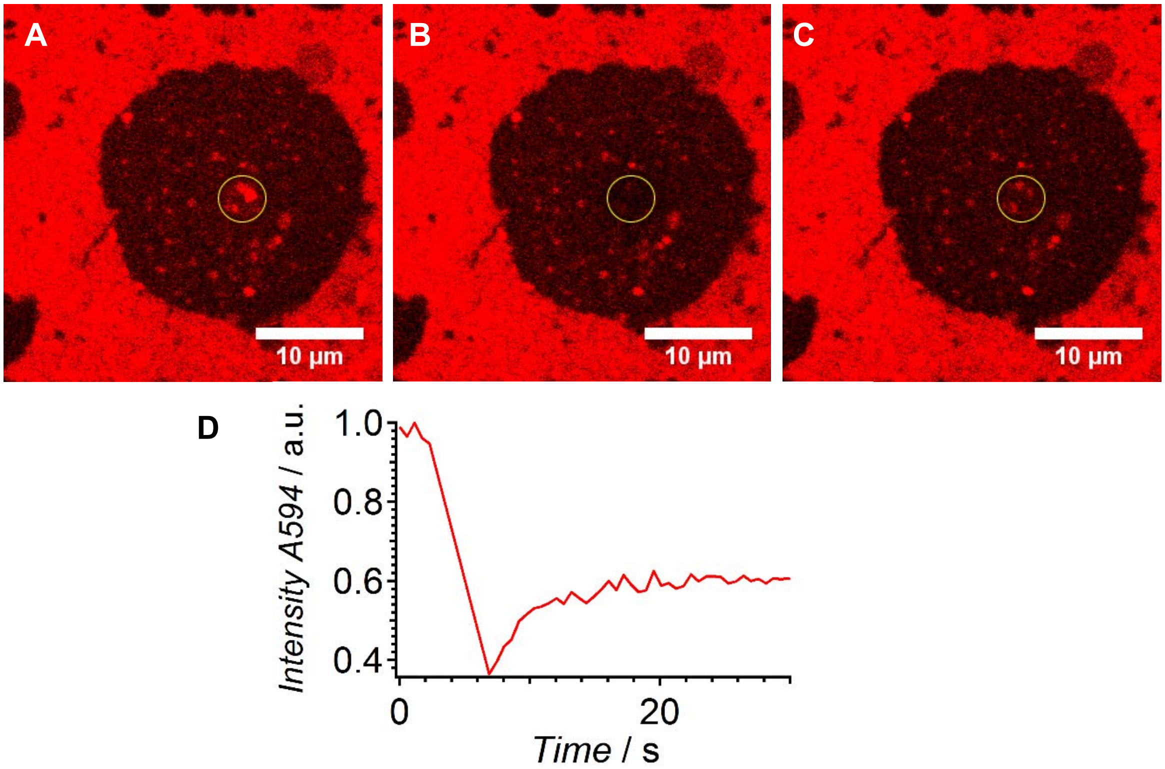

Fig. S10 | FRAP measurement of fused and docked vesicles. A) Vesicles (LUVs) in the ROI (yellow circle) are visible as bright red spot before starting the FRAP-experiment. B) Bleaching of the vesicles led to a complete loss of fluorescence intensity. C) Two of the three vesicles showed recovery. The largest vesicle from image (A), however, shows no recovery because this was only a docked or adhered vesicle. D) Mean fluorescence intensity collected from the entire circle during the time course of the experiment.

In figure S12, all FRAP measurements for the determination of diffusion coefficients have been compiled in a boxplot. The mean diffusion coefficient of SLBs on PDMS was calculated to be around 0.6-0.9 $\mu m^2\ s^{-1}$, which agrees well with the expected mobility of fluid lipids in the membrane.

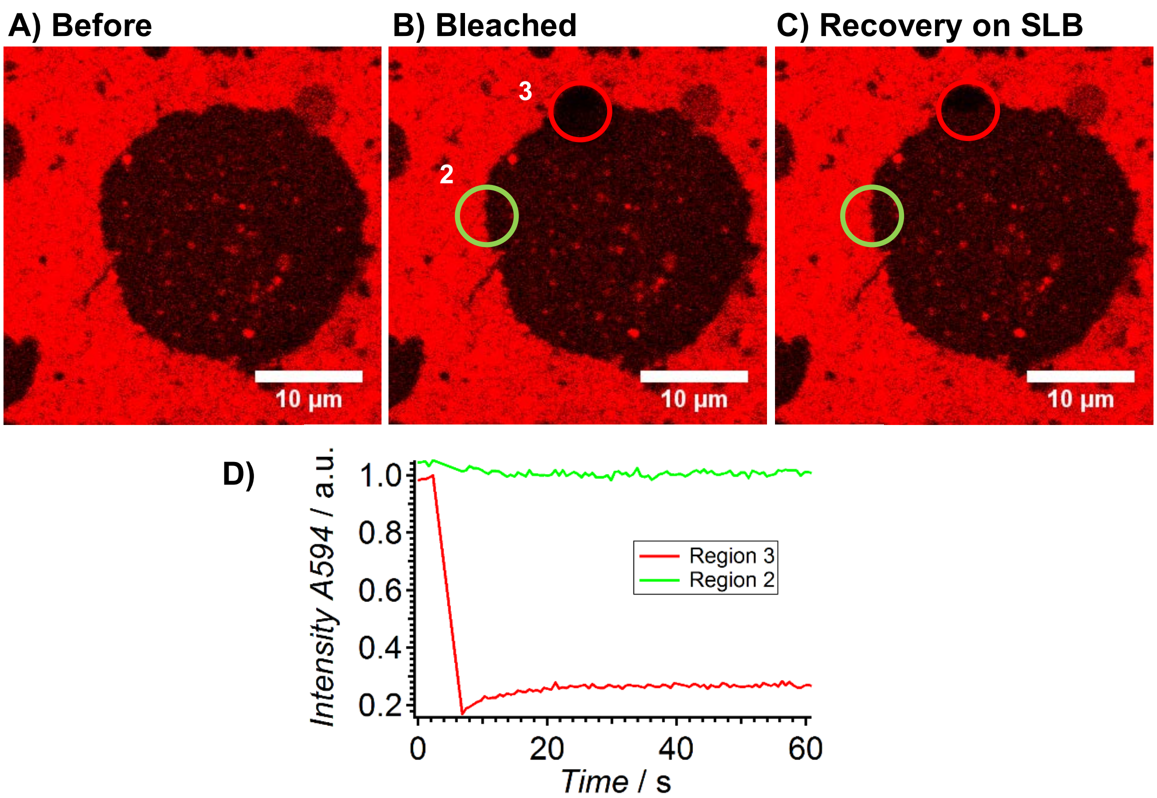

Fig. S11 | FRAP on the edge of a SLB patch. A)-C) Images taken during the FRAP measurement. The red circle shows the bleaching spot and the green circle serves as a reference. D) Fluorescence intensity of the bleached (Region 3) and reference area (Region 2).

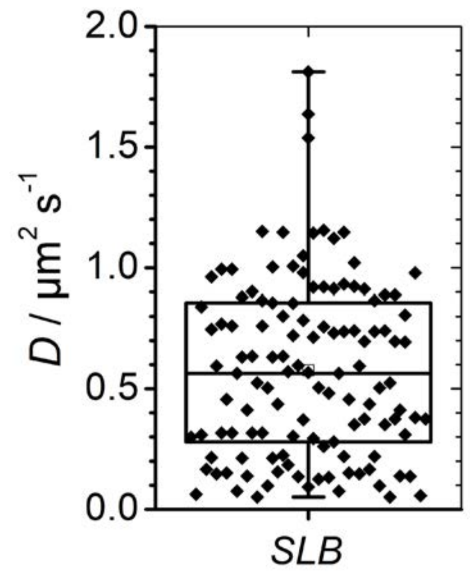

Figure S12 | FRAP at SLBs. Diffusion coefficient of SLBs on PDMS. Box: 25-75 percentile. Whiskers include all data points (black diamond squares). Bar in the box: Median. White Square: Mean value.

Section 4: Lipid mixing of LUVs with adhered GUVs

Experiment 1: Low membrane tension

In experiment 1 the GUVs were slightly adhered to the functionalized glass surface to generate a low pre-stress. The adjusted low membrane tension on the GUVs serves as a reference for highly tensed membranes to compare the docking and fusion efficiency of LUVs. Typical membrane tensions in biological cells are in the range of 0.01 mN/m to 0.3 mN/m.[2] Therefore, the membrane tension of a total of 13 GUVs was adjusted to a similar range between 0.17 mN/m and 1.2 mN/m. In the figures S13-S16, all measured GUVs are shown in cross-sectional images. In order to categorize docking and fusion of LUVs on each GUV, the image on the left side is composed as a two-channel image for both dyes to visualize docking of LUVs to the freestanding GUV-membrane. On the right side of figures S13-S16 the cross-sectional views of the channel for the LUV dye are shown. Adhesion of LUVs to the sample surface was found to occur in all measurements and the fluorescence intensity of the adhered LUVs served as a reference (100 %) for the docked LUVs on the GUVs.

[2] Kozlov, M. M.; Chernomordik, L. V., Membrane tension and membrane fusion. *Curr. Op. Struct. Biol.* **2015,** *33*, 61-67.

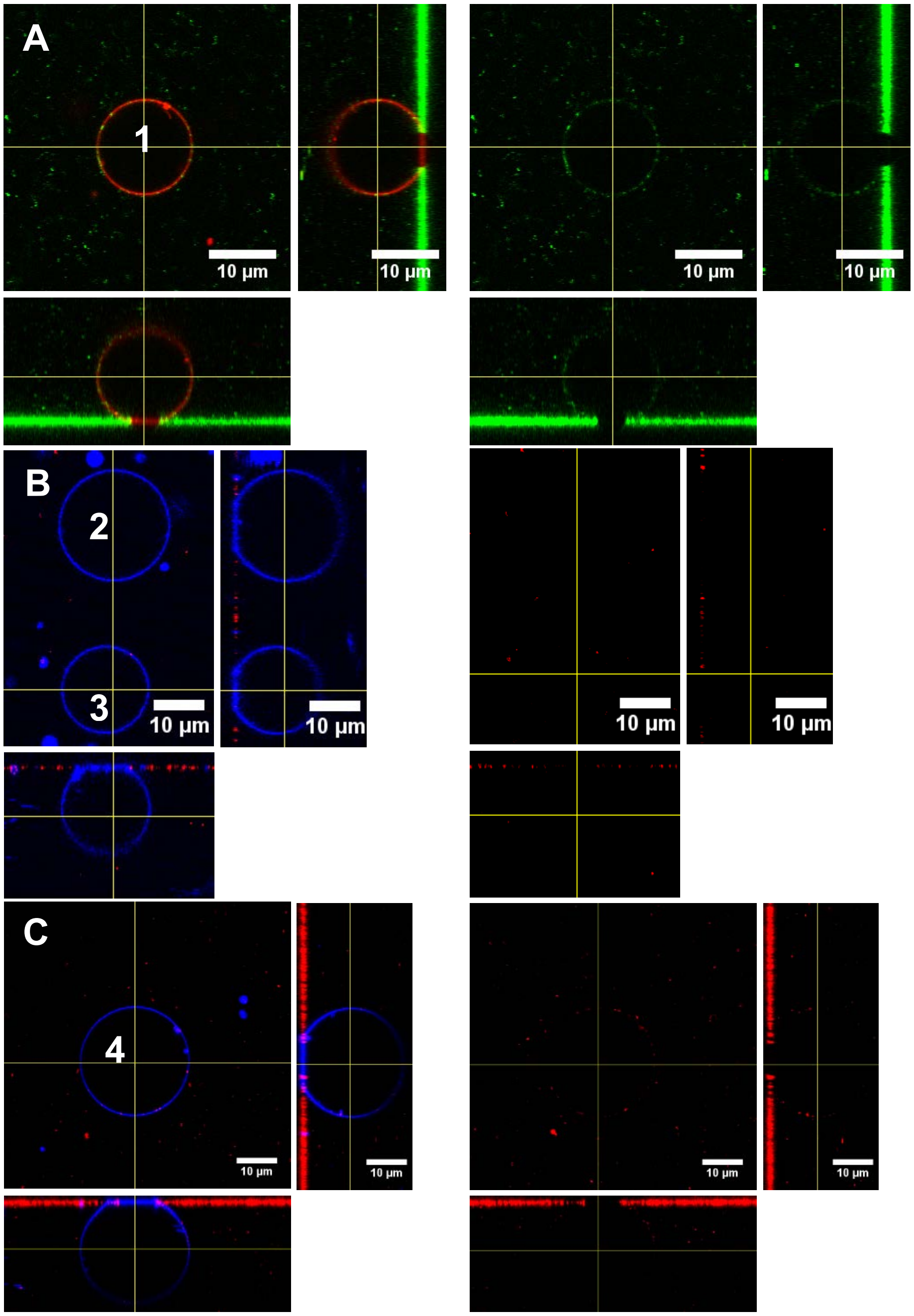

D

GUV No.	R_i / µm	$\widetilde{R}_v$ / µm	$\Delta A/A_0$ / %	σ / mN m^{-1}
1	3.2	7.0	0.3	0.30
2	4.6	10.8	0.21	0.23
3	4.3	8.8	0.41	0.44
4	7.1	13.5	0.5	0.56

Figure S13 | Low tension. GUVs with low membrane tension display only few docked LUVs. A)-C) Left: Two channel images show GUVs (A390) and LUVs (A488 or A594). Right: Only one channel for the LUV dye. Fluorescently labeled lipids in the GUV/LUV-membrane: A) TR (red) / A488 (green), B)-C) A390 (blue) / A594 (red). D) Table of measured radii, area changes $\Delta A/A_0$ and corresponding membrane tension σ.

In figure S13, four slightly adhered GUVs (1-4) with membrane tensions between 0.3 mN/m to 0.56 mN/m are shown that exhibit nearly no docked LUVs. GUVs 5-7 are shown in figure S14. Docking of LUVs to the GUV-membrane occurred mainly on GUV 5, which shows a larger adhesion radius compared to the other GUVs. The calculated membrane tension of 1.2 mN/m for GUV 5 is significantly higher compared to that of the other GUVs. The adhesion site of GUV 5 shows no fluorescence intensity originating from the LUV dye. Therefore, lipid mixing with the membrane of GUV 5 can be excluded. In figure S14 A and B, the surrounding solution of the adhered GUVs contained a higher concentration of LUVs compared to image C. The GUVs 8-12 in figure S15 were incubated with LUVs for 50 minutes to see whether LUVs dock to the membranes over a prolonged time more frequently. Even after such a long time an increased docking and fusion of the LUVs to GUVs with membrane tensions lower than 0.51 mN/m could not be observed.

In figure S16, GUV 13 is shown with many docked LUVs at the freestanding membrane. Compared to GUV 7 in figure S14 C with a membrane tension of 0.78 mN/m the docking on GUV 13 is significantly increased, which could be related to a higher LUV concentration in the surrounding solution. Already at this moderate tension, fusion of LUVs with GUV 13 can be monitored. The lipid mixing between the vesicles after fusion leads to the diffusion of the fluorescently labeled lipids into the adhesion area of the GUV. After the addition of the LUVs, GUV 13 exhibited a larger adhesion radius.

In summary, the docking probability of LUVs is increased at membrane tensions above 0.8 mN/m (Fig. S16 and S14 A). Generally, membrane tensions below 1.2 mN/m on adhered GUVs do not lead to a measureable amount of fusion.

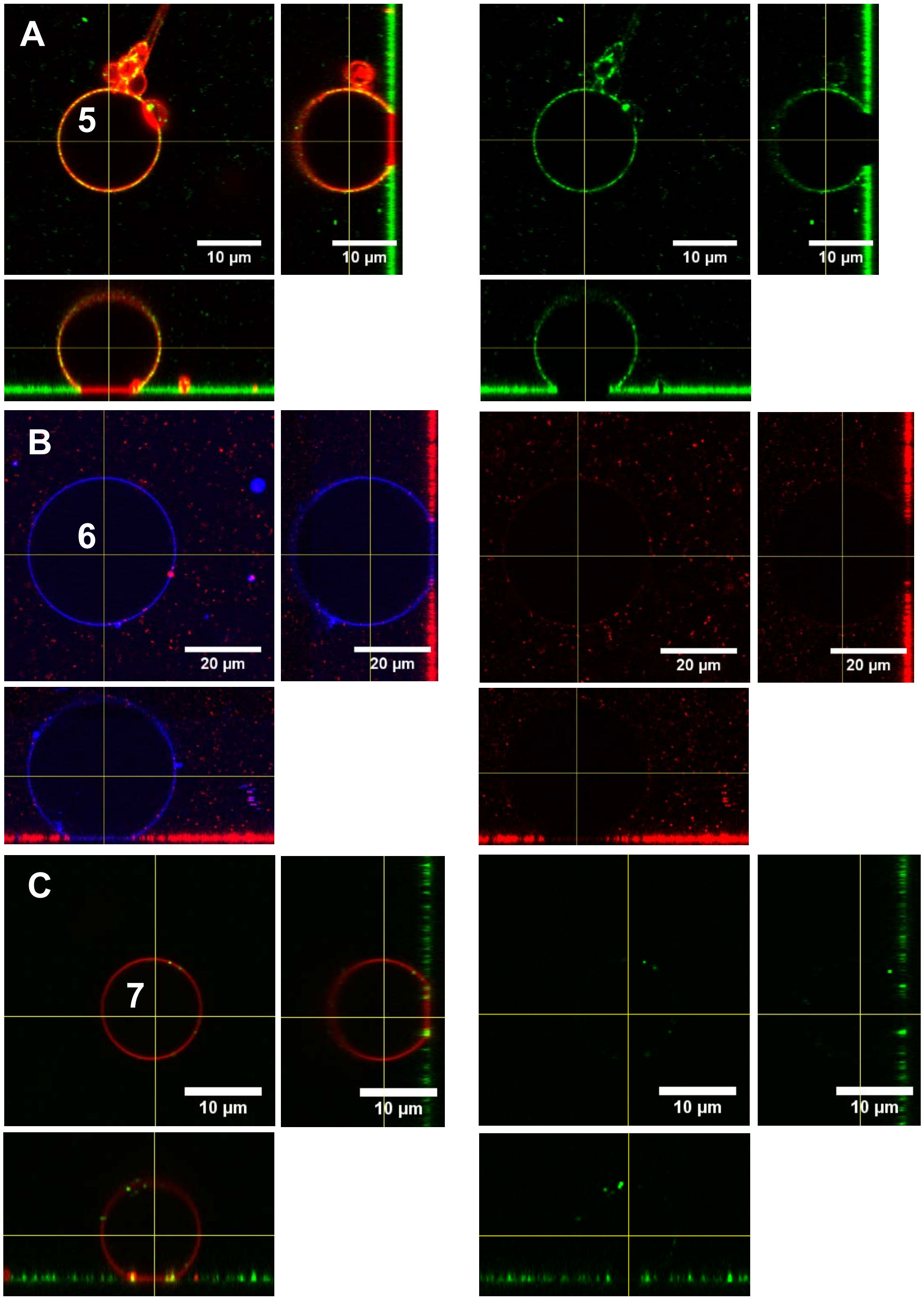

D

GUV No.	R_i / µm	$\tilde{R}_v$ / µm	$\Delta A/A_0$ / %	σ / mN m^{-1}
5	4.7	7.9	0.85	1.2
6	7.9	18.7	0.18	0.2
7	3.6	6.5	0.64	0.78

Figure S14 | Low tension. A)-C) GUVs with a low membrane tension and a few docked LUVs. Fluorescently labeled lipids in the GUV/LUV-membrane: TR (red) / A488 (green), A390 (blue) / A594 (red). D) Table of measured radii, area changes and corresponding membrane tensions.

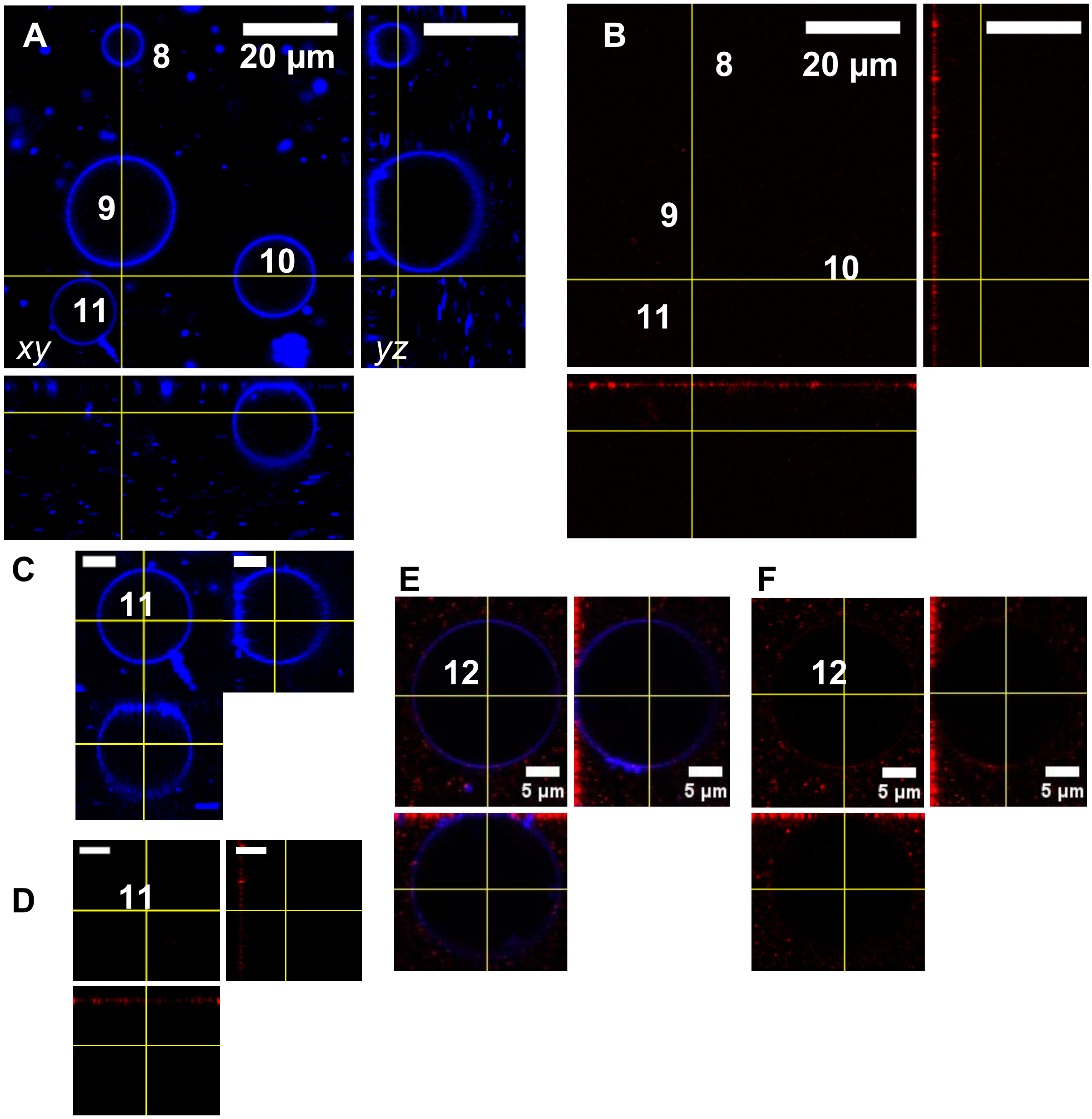

G

GUV No.	R_i / µm	$\tilde{R}_v$ / µm	$\Delta A/A_0$ / %	σ / mN m^{-1}
8	2.2	4.3	0.46	0.51
9	5.6	12.5	0.26	0.27
10	4.2	8.7	0.34	0.36
11	2.6	6.4	0.19	0.21
12	4.0	10.7	0.13	0.17

Figure S15 | Low tension. Slightly adhered GUVs with low membrane tension exhibit no LUV fusion. A), C), E) Cross-sectional view of the GUV-membrane (blue) containing A390. B), D), F) The red channel of the LUV dye A594 shows no fluorescence on the GUVs but on the substrate surface. A few LUVs dock to the membrane of GUV 12. G) The table shows the measured radii and area changes of the GUVs and lists the calculated membrane tensions.

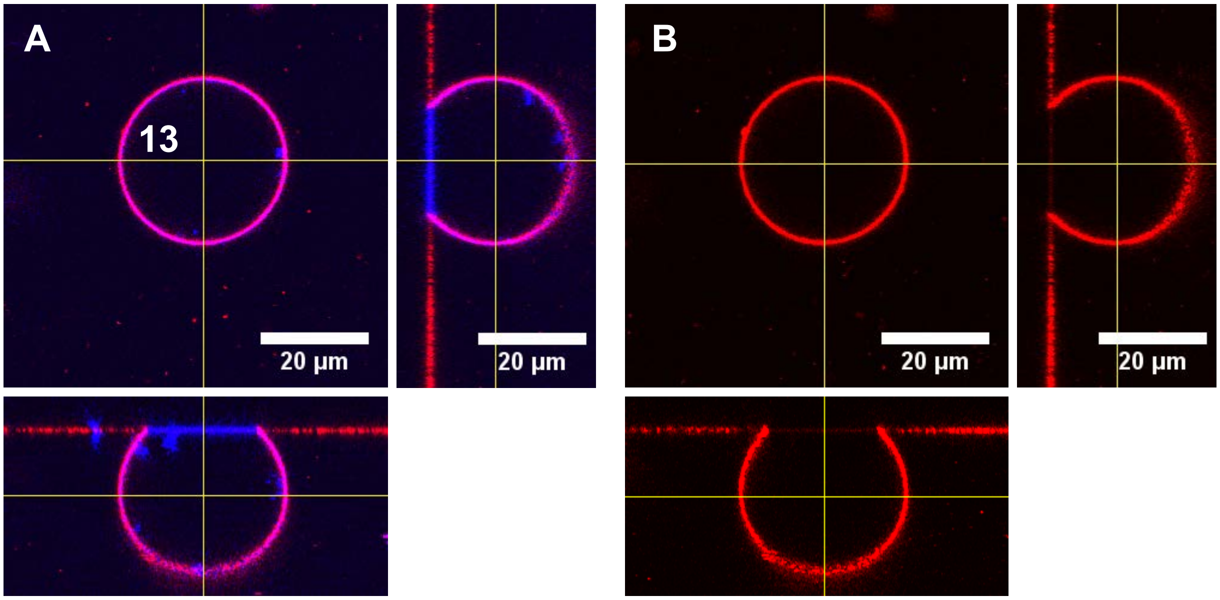

C

LUV-addition	R_i / µm	$\tilde{R}_v$ / µm	$\Delta A/A_0$ / %	σ / mN m^{-1}
before	8.5	15.1	0.67	0.83
after	10.3	15.1	1.5	2.6

Figure S16 | Moderately tensed GUV. A) Emission of both dyes from the GUV (blue) and the LUVs (red) are shown in an overlay image and the corresponding cross-sectional images on the right and bottom. B) The red channel documents a high docking rate at the freestanding GUV-membrane and a slightly lower red fluorescence intensity at the adhesion area of the GUV is indicative of fusion. C) The table shows the measured radii and area changes of GUV 13 for the calculation of membrane tension.

Experiment 2: Elevated membrane tension

Comparable membrane tensions to those occurring in cells do not lead to a significant increase of vesicle fusion to pre-stressed GUVs as it is described in experiment 1. Therefore, in experiment 2 GUVs with membrane tensions from 1.1 mN/m to 8.6 mN/m served as target membranes for the investigation of the docking and fusion efficiency of LUVs. We used an elevated Mg^{2+} concentration to foster adhesion as the driving force for stretching.

In figures S17-S19 the GUVs 14-23 with an elevated membrane tension are shown after the incubation of LUVs. GUV 14 shown in figure S17 has the largest membrane tension of 8.6 mN/m of all measured GUVs, close to lysis tension. The two-channel image on the left side of the figures shows both fluorescently labeled lipids and on the right-side the channel for the LUV dye is shown (GUV labeled with ATTO A390, LUVs with ATTO A594 or A488, respectively). All highly-tensed GUV-membranes reveal a large number of docked LUVs. At the adhesion site of the tensed GUVs, the fluorescence intensity of the LUV dye was increased indicative of fusion with the GUV-membrane. To prove that the fluorescently labeled dye originating from LUVs can diffuse freely in the GUV-membrane FRAP experiments were carried out at the adhesion site as described in section 5.

In figures S19 C-E two touching GUVs (22 and 23) are shown. After fusion with LUVs the LUV dye A594 diffused into the connection site between the two GUVs. The membrane tensions are 1.5 mN/m for GUV 22 and 2.3 mN/m for GUV 23 (Figure S19 F). An area scan from figure S19 D is shown in the graph (E) which shows the fluorescence intensity of the GUV dye A390 and the LUV dye A594. No docked LUVs appear in the contact zone between the two GUV-membranes. The mean fluorescence intensity of the LUV dye A594 is increased in both GUVs. Especially, at the connection site the fluorescence intensity is higher which could be due to the presence of two lipid bilayers containing the fluorescently labeled lipid originating from fused LUVs. The error for the measurement of the vesicle radii for the GUVs 22 and 23 is assumed to be slightly higher because of the non-spherical shape of the GUVs.

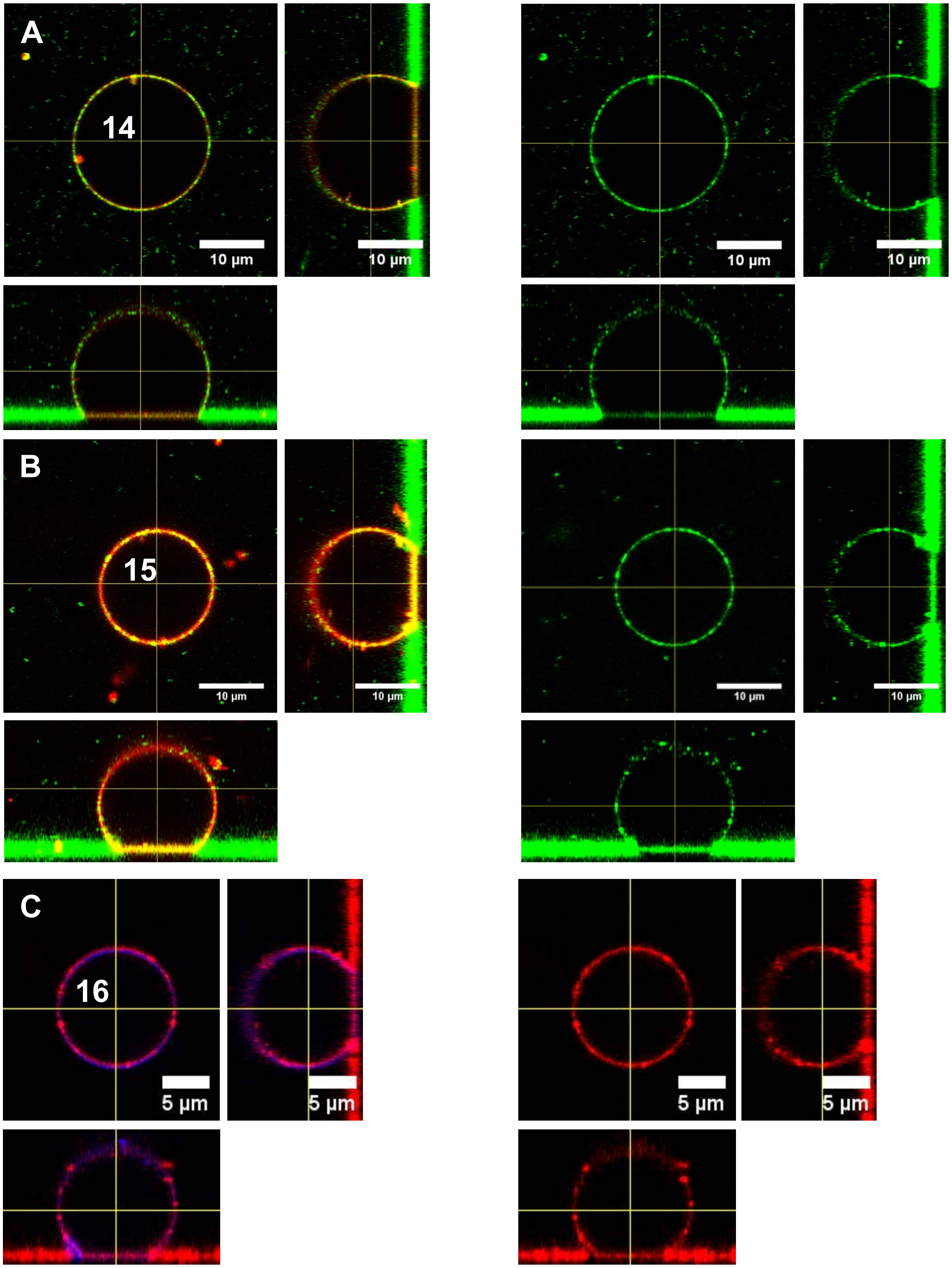

D

GUV No	R_i / µm	$\widetilde{R}_v$ / µm	$\Delta A/A_0$ / %	σ / mN m^{-1}
14	8.8	10.6	3.9	8.6
15	6.5	9.0	2.1	4.0
16	4.1	6.1	1.4	2.4

Figure S17 | Elevated tension. Strongly adhered GUVs with high membrane tension reveal LUV fusion. Fluorescently labeled lipids in the GUV/LUV-membrane: TR (red) / A488 (green), A390 (blue) / A594 (red). A)-C) Cross-sectional images of GUVs reveal high amount of docked and fused LUVs at the GUV-membrane. D) The table shows the measured radii and area changes of the GUVs 14-16 and the calculated membrane tensions.

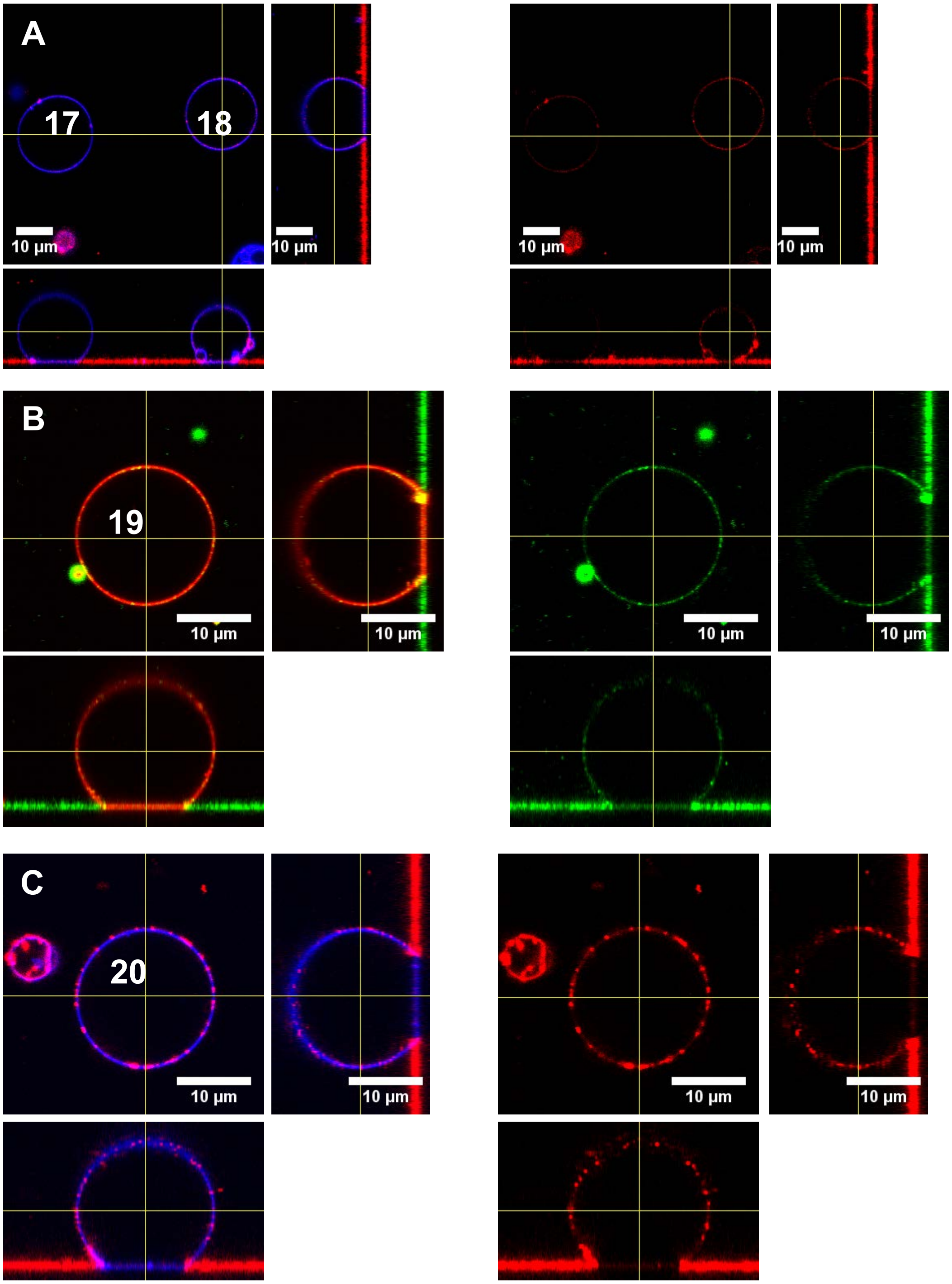

D

GUV No.	R_i / µm	$\widetilde{R}_v$ / µm	$\Delta A/A_0$ / %	σ / mN m^{-1}
17	6.0	10.2	0.83	1.1
18	7.3	9.7	2.5	5.0
19	5.9	9.2	1.1	1.7
20	5.8	9.2	1.1	1.7

Figure S18 | Elevated tension. Strongly adhered GUVs with high membrane tension reveal LUV fusion (right). Fluorescently labeled lipids in the GUV/LUV-membrane: TR (red) / A488 (green), A390 (blue) / A594 (red). A)-C) Cross-sectional images of GUVs reveal high amount of docked and fused LUVs at the GUV-membrane. D) The table shows the measured radii and area changes of the GUVs14-16 and the calculated membrane tensions.

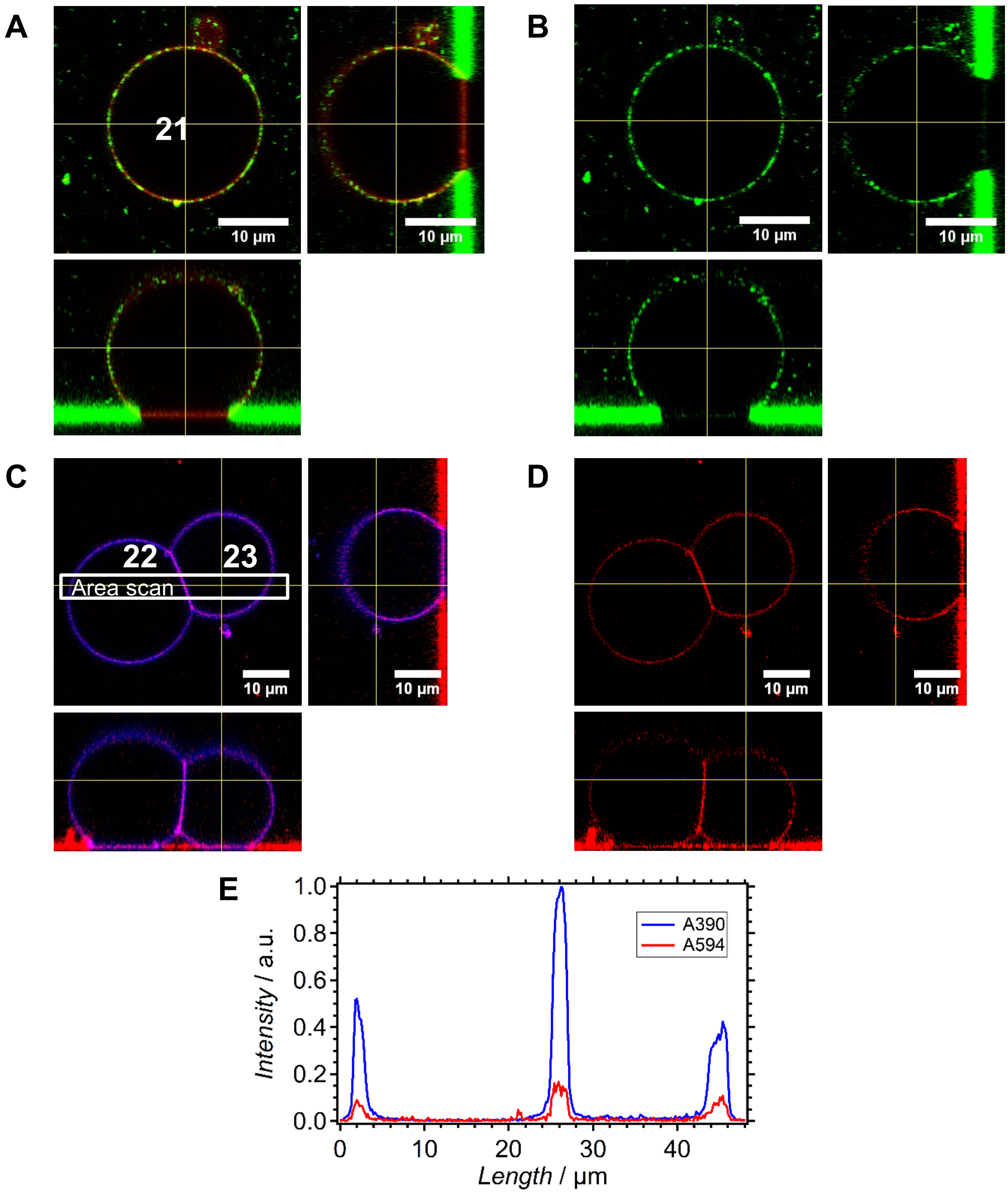

F

GUV No.	R_i / µm	$\tilde{R}_v$ / µm	$\Delta A/A_0$ / %	σ / mN m^{-1}
21	7.0	10.8	1.2	2.0
22	8.1	13.2	1.0	1.5
23	7.5	11.6	1.4	2.3

Figure S19 | Connected GUVs adhering in near vicinity on the substrate. A) The two-channel image shows the two adjacent GUVs (blue) and the area scan in the white box. B) The red channel of the LUV dye A594 shows a fluorescence intensity homogeneously distributed all over the GUV membrane. C) The graph represents the area scan from (A) for the mean fluorescence intensity of both lipid dyes. The LUVs fused with the GUV-membrane and thus the LUV dye is present also at the connection site between the GUVs. D) The table lists the measured radii, area changes and corresponding membrane tensions.

Section 5: FRAP measurements

FRAP measurements at the adhesion site of GUVs

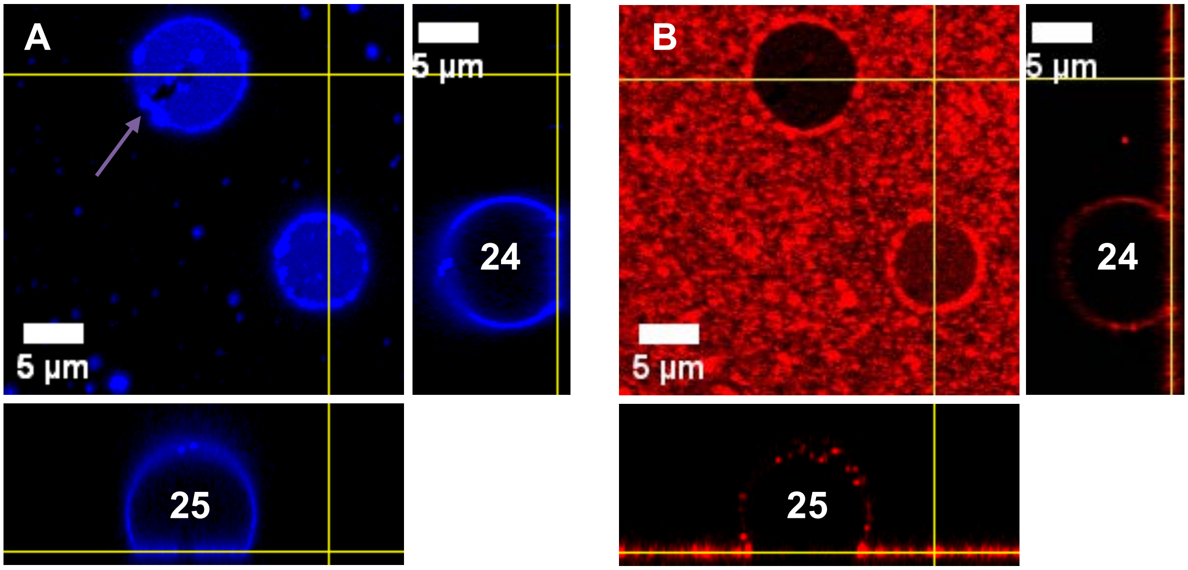

C

GUV No.	R_i / µm	$\widetilde{R}_v$ / µm	$\Delta A/A_0$ / %	σ / mN m^{-1}
24	3.9	5.2	2.4	4.8

Figure S20 | Comparison of tensed and ruptured GUV-membranes. A) The adhesion area of two different GUVs is shown in the *xy*-plane. GUV 25 ruptured at the adhesion site, whereas GUV 24 is still intact. B) LUV docking and fusion appeared on GUV 4 more frequently because of the high fluorescent intensity originating from the LUV dye A594. C) The table compiles the measured radii and calculated area changes as well as membrane tensions for GUV 24.

The FRAP-measurement performed on GUV 24 is shown in figure S21 and provides a diffusion coefficient of approximately 1.3 µm^2 s^{-1} with an immobile fraction of around 40%. In figures S21 (F-I) the FRAP-measurements of adhered LUVs on the surface are also shown. Bleaching of merely docked LUVs on the substrate surface yields no fluorescence recovery. Thereby we confirm that the adhered LUVs did not spread on the substrate surface and thus fuse with the GUV-membrane. In figure S21 a FRAP-measurement carried out on the adhesion site of GUV 14 formed with the substrate is shown. Bleaching with a LASER (488 nm) led to a loss of fluorescence intensity within the green ROI. Ten seconds after the bleaching, the recovery reached 60% of the initial fluorescence intensity. For the fluorescently labeled dye A488 at the adhesion site, a diffusion coefficient of 0.8 µm^2 s^{-1} was calculated and an immobile fraction of about 17% was found. In figures S21 F-J the edge of the adhesion area was bleached and a recovery was detected, whereby the fluorescence intensity recovered to around 60% of its initial value.

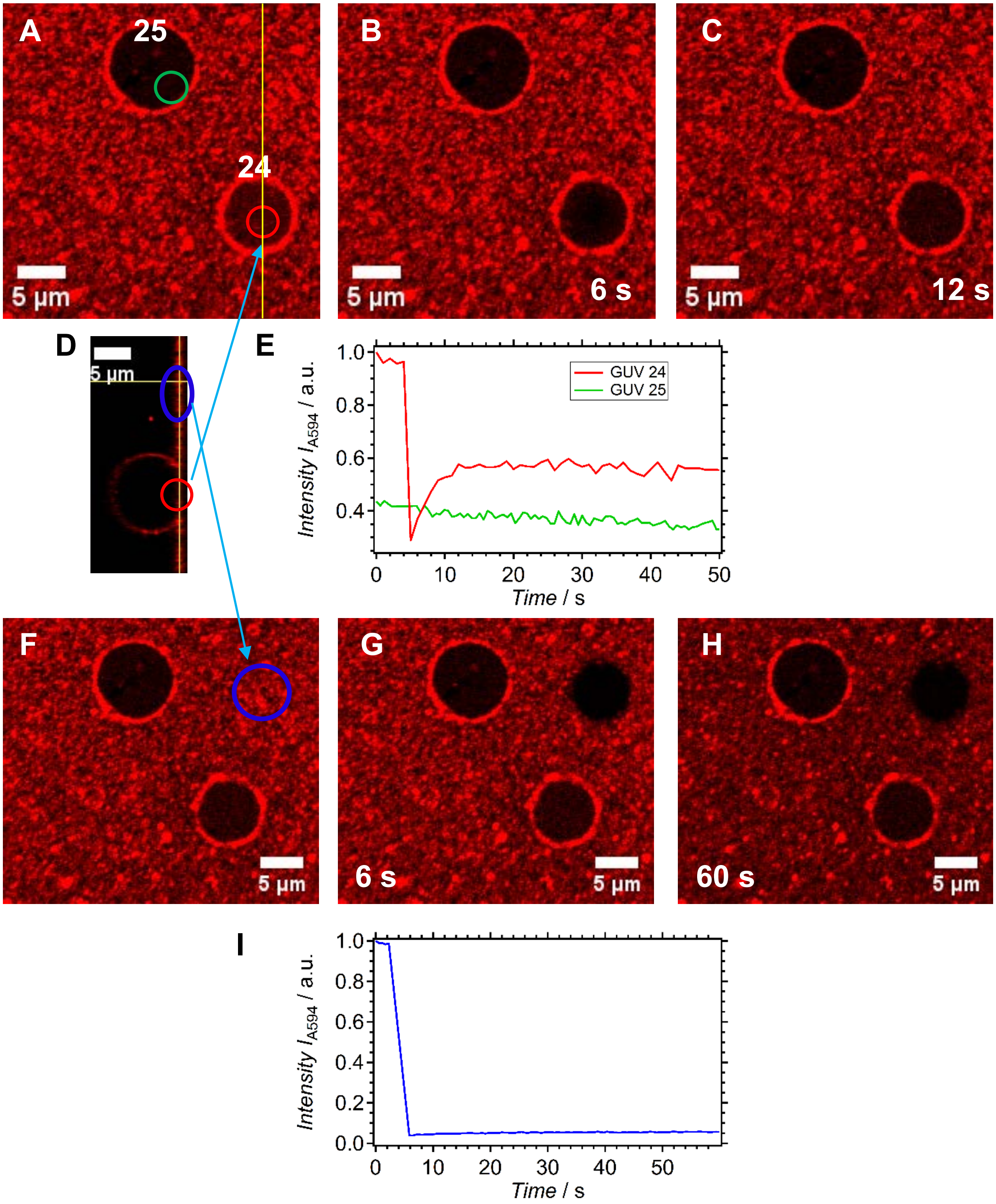

Figure S21 | FRAP-measurement at the adhesion site of the GUVs from figure S20. A) The red circle shows the area chosen for bleaching of the GUV 24 with a LASER (561 nm) and the green circle serves as the reference area. B) The image was taken directly after the bleaching of the red circle area depicted in (A). C) Seven seconds after bleaching the recovery was finished, with around 40% of immobile fraction. D) The cross-sectional (*yz*-plane) image shows bleaching areas on the substrate surface. E) The graph shows the recovery of the mean fluorescence intensity at the red ROI of GUV 24. F-I) The bleached LUVs on the surface shows no recovery after 60 s.

The diffusion coefficient obtained from the FRAP-measurement shown in figure S21 A-E is around 1.3 $\mu m^2 s^{-1}$. For GUV 15 in figure 22 F-J a diffusion coefficient could not be determined because the bleached area was at the edge of the adhesion site so that docked LUVs were bleached too.

In summary, the FRAP-measurements shown in figures S21 and S22 confirm lipid mixing of the adhered GUVs and added LUVs. Moreover, LUVs do not spread on the surface by forming a lipid bilayer that could possibly fuse with the adhered GUV membrane.

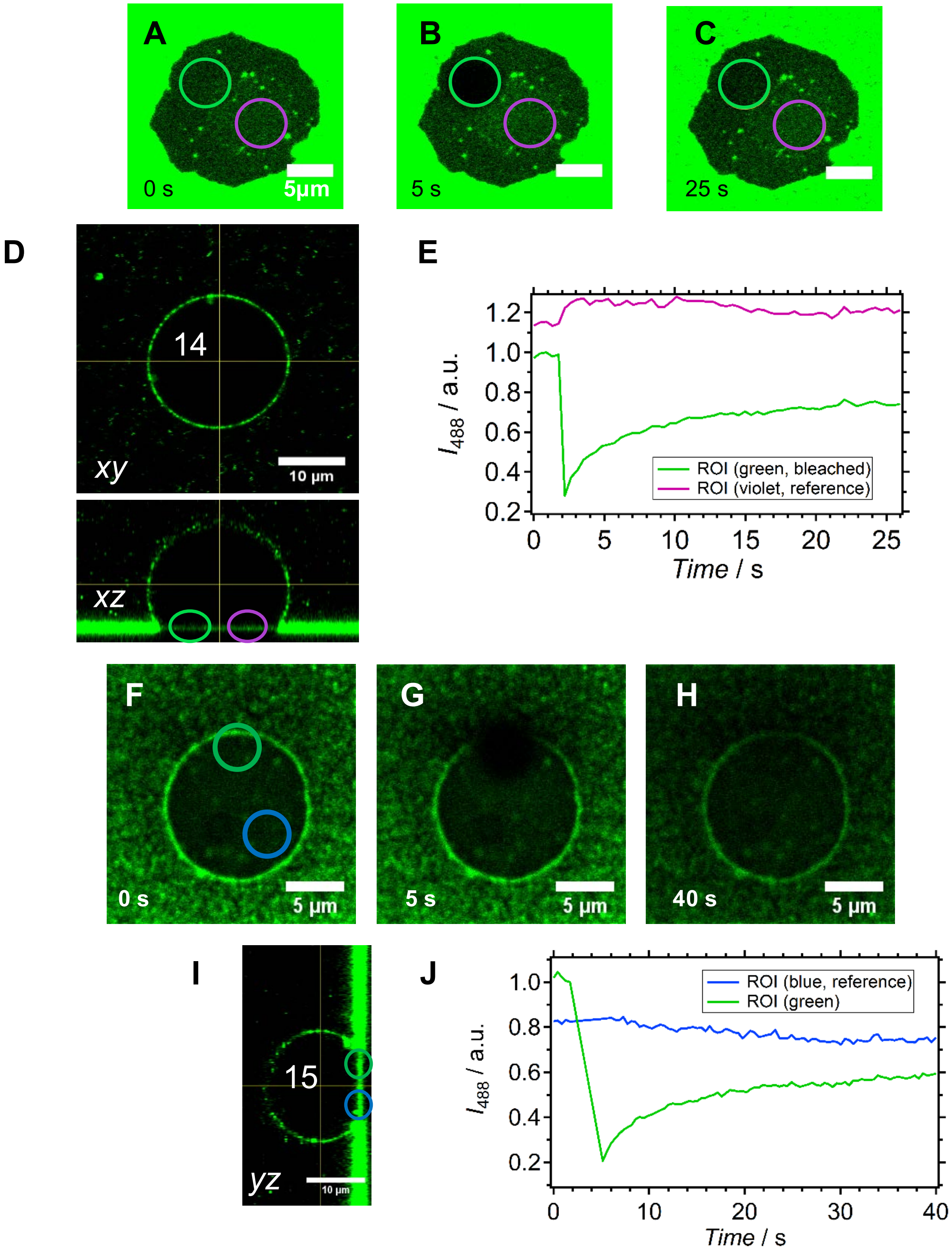

Figure S22 | FRAP measurement at the adhesion area of GUV 14. The brightness of fluorescence intensity in the images (A)-(C) was increased to visualize the fluorescence intensity before (A), after bleaching (B) and after recovery (C). D) The cross-sectional view shows the bleaching (green circle) and reference area (purple circle). E) The graph shows the mean fluorescence intensity for both circles with the purple circle as a reference (100%). F-H) Images of a FRAP-measurement at the edge of the adhered membrane of GUV 15. G) The edge of the adhered GUV-membrane is bleached. H) The fluorescently labeled lipid A488 recovers to 60% of its initial intensity. I) The green ROI in the cross-sectional view of GUV 15 shows the bleaching area and the blue ROI serves as the reference. E) Mean fluorescence intensity of the bleached ROI (green) and intensity from the reference ROI area (blue) are shown.

Section 6: Content mixing of LUVs with adhered GUVs

If two membranes fuse, they eventually share the same content. The content mixing experiment, illustrated in figure S23, was carried out by adding LUVs, containing the water-soluble dye ATTO® 488, to the adhered GUVs. An increased fluorescence intensity inside the GUV reveals that the LUVs fuse fully with the GUV-membrane. Merging of vesicle membranes is mediated by the use of SNARE proteins as described in experimental section.

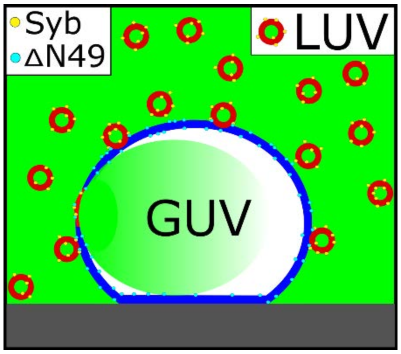

Figure S23 | Content mixing. Mixing of contents was achieved by the fusion of LUVs (red) to the adhered GUV (blue) so that the content of the LUVs is poured inside the GUV.

In figure S24 three GUVs with different sizes and adhesion areas are shown in a *xy*-plane picture and a corresponding cross-sectional view on the right and bottom. After incubation of LUVs containing the water soluble green fluorescent dye ATTO A488 with adhered GUVs, the GUVs 27 and 28 show a green fluorescence from the lumen, while GUV 26 shows nearly no intensity emitted from the inside (figure 24 C+D). The corresponding membrane tension of each GUV is given in the table S1.

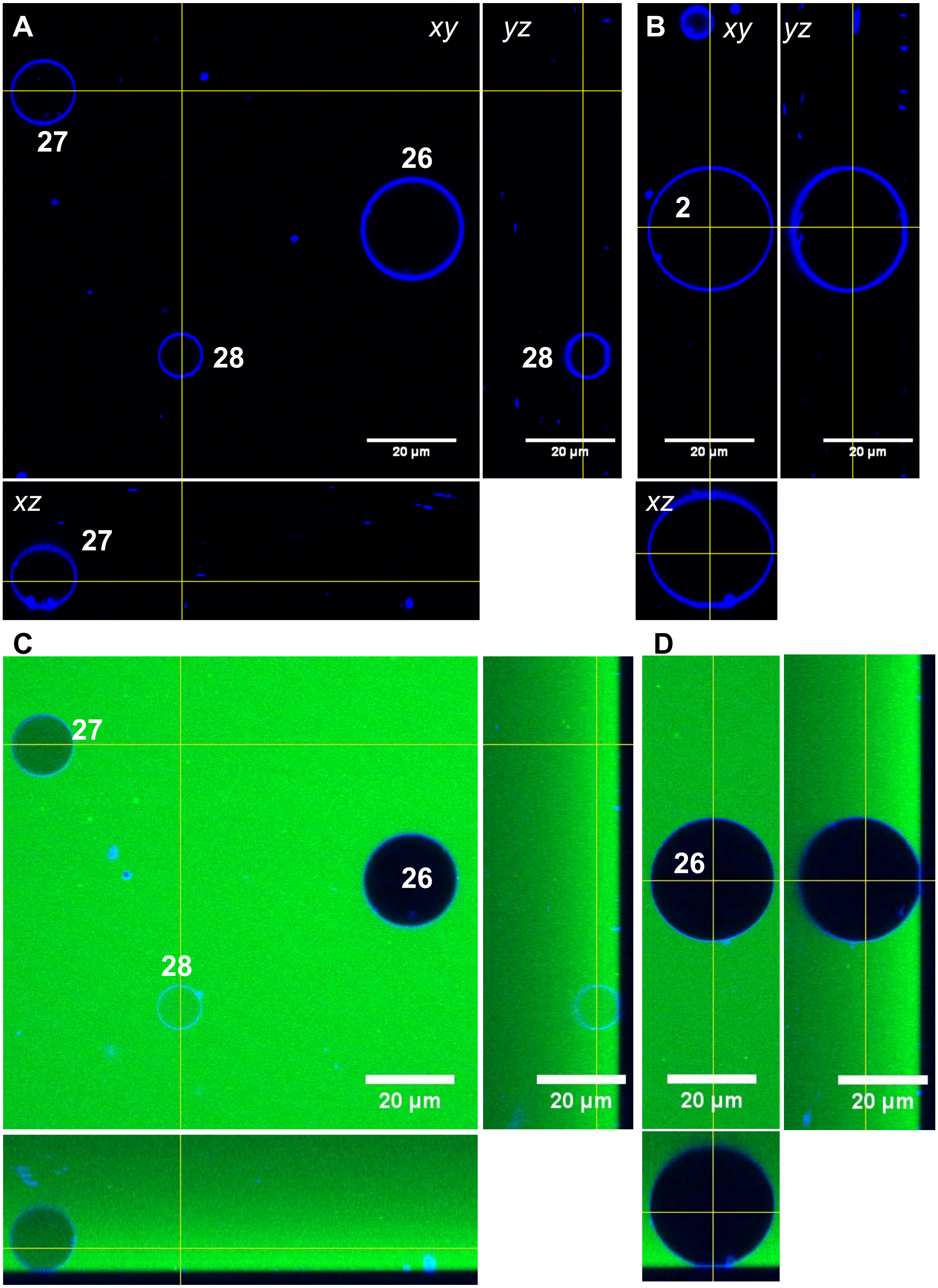

Figure S24 | Content mixing assay. A) Three GUVs 26-28 are shown. B) The cross-sectional image for GUV 26 is shown. C) After LUV incubation GUVs 27 and 28 contained the green fluorescent dye ATTO® 488. D) The largest GUV with the smallest membrane tension shows no fluorescence intensity emitted from the lumen.

Table S1 | Membrane tension of GUVs shown in figure S24.

GUV No.	R_i / µm	$\tilde{R}_v$ / µm	$\Delta A/A_0$ / %	σ / mN m^{-1}
26	4.30	13.98	0.05	0.12
27	3.35	7.31	0.28	0.29
28	2.88	5.01	0.73	0.94

The FRAP-measurement shown in figure S25 shows that the GUV content can be bleached by the LASER without recovery confirming that the GUVs are not leaky. To further exclude diffusion of the dye ATTO® 488 from the surrounding buffer solution into the GUV a control sample without SNAREs in neither the GUVs nor the LUVs is shown in figure S26. Even strongly adhered GUVs with a high membrane tension do not contain any fluorescence intensity so that non-specific fusion and diffusion of the water-soluble LUV dye through the GUV-membrane can be excluded.

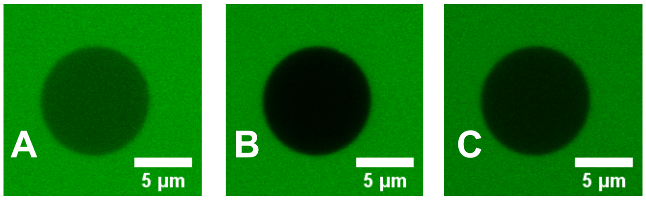

Figure S25 | Content mixing - FRAP measurement. The images were taken before (A) and after (B) bleaching of the GUV content at 6 s. Image (C) was the last one more than 300 s after the GUV content was bleached.

Control measurements in the absence of SNAREs

Content mixing in the absence of SNAREs due to leaky GUVs was exclude by preparing samples with the water soluble fluorescent dye ATTO 488® carboxy (green). Fusion of LUVs, containing the water-soluble fluorescent dye inside, with the adhered GUVs was not observed (Figure S26). Also, LUVs containing synaptobrevin did not fuse with an adhered GUV (Fig. S26 A). For the two GUVs 1 and 2 from figure S26 the corresponding membrane tension is listed in the table (C). Content mixing in the absence of the ΔN49-complex at the adhered GUV (Fig. S26 A) or in the absence of both synaptobrevin orthe ΔN49 complex was not observed even for highly tensed membranes (Fig. S26 B+C).

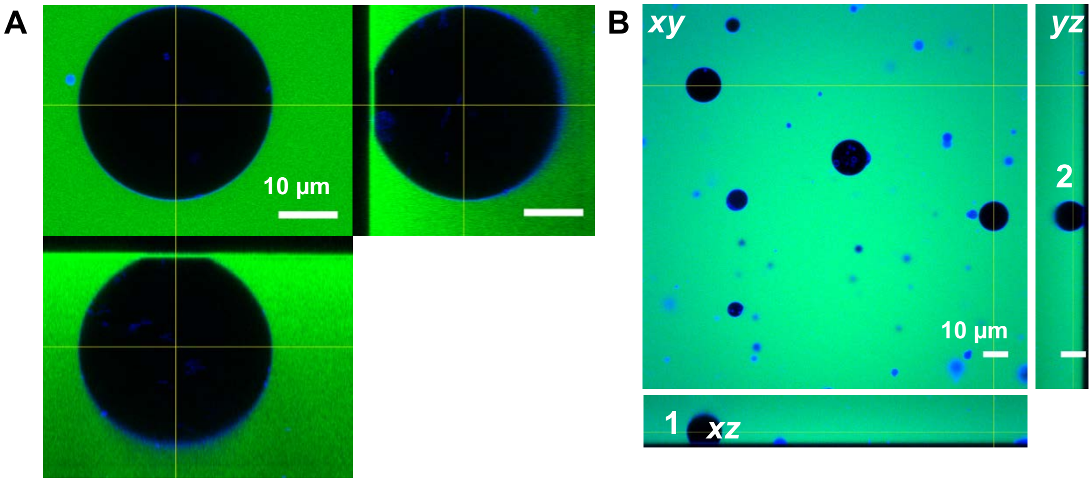

C

GUV No.	R_i / µm	$\tilde{R}_v$ / µm	$\Delta A/A_0$ / %	σ / mN m^{-1}
1	5.7	7.2	3.2	6.9
2	4.0	5.9	1.4	2.3

Figure S26 | Content mixing without SNAREs. A) Content mixing assay with synaptobrevin at the incubated LUVs but without the reconstituted ΔN49-complex in the adhered GUVs. The green fluorescent dye inside the LUVs did not diffuse into the GUV confirming that the vesicles are not leaky. B) GUVs in the absence of SNAREs shows no content mixing after addition of LUVs filled with a green fluorescent dye. The cross-sectional view shows that the GUVs strongly adhere on the surface generating a highly-tensed membrane but still do not show fusion. C) The table compiles the measured radii, area changes and the corresponding membrane tension of the GUVs 1 and 2 from B.

Section 7: Dynamic Light scattering – LUV size distribution

LUVs were produced by the detergent dilution method as described in the materials and methods section of the paper. In figure S27, the typical diameter size distribution is shown measured by dynamic light scattering in buffer solution.

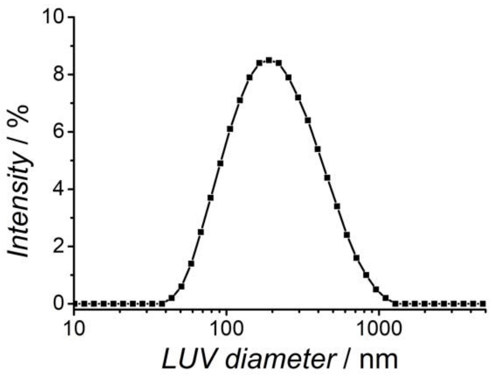

Figure S27 | Size distribution of LUVs measured by dynamic light scattering. The average size of the LUVs is around 200 nm in diameter.
